# In vivo biocompatibility assessment of 3D printed bioresorbable polymers for brain tissue regeneration. A feasibility study^☆^

**DOI:** 10.1016/j.reth.2024.10.004

**Published:** 2024-10-23

**Authors:** Julien Clauzel, Nina Colitti, Maylis Combeau, Wafae Labriji, Lorenne Robert, Adrien Brilhault, Carla Cirillo, Franck Desmoulin, Isabelle Raymond-Letron, Isabelle Loubinoux

**Affiliations:** a ToNIC, Toulouse NeuroImaging Center, Université de Toulouse, Inserm, UPS, Toulouse, France; b LabHPEC, Université de Toulouse, ENVT, Toulouse, France; c Institut Restore, Université de Toulouse, CNRS U-5070, EFS, ENVT, Inserm U1301, Toulouse, France

**Keywords:** 3D printing, Scaffold, Central nervous system, Tissue bioengineering, MRI, Brain injury

## Abstract

**Introduction:**

The limited capacity of brain tissue to regenerate after acute injury, hampered by cell death, edema and inflammation, has led to an interest in promising and innovative approaches such as implantable regenerative scaffolds designed to improve brain plasticity. Leveraging the capabilities of bioprinting, these scaffolds can be tailored to match the intricate architecture of the brain.

**Methods:**

In this methodological study, we performed in vivo biocompatibility assessments after a brain lesion on three distinct bioeliminable or bioresorbable materials: Poly(ethylene glycol) diacrylate (PEGDA), Polycaprolactone (PCL) and a PEGDA mixed with gelatin methacrylate (PEGDA-GelMA).

**Results:**

A scaffold with a complex shape was printed with patterns, spatial resolution and porosity adapted to cerebral cortex reconstruction. In vivo evaluations were complemented by behavioral monitoring, affirming the safety of these materials. High-resolution T2 MRI imaging effectively captured scaffold structures and demonstrated their non-invasive utility in monitoring degradability. ASL MRI imaging quantified cerebral blood flow and was positively and significantly correlated with lectin immunofluorescent labeling. It may be used to non-invasively monitor progressive revascularization of implants.

PEGDA produced an intense foreign-body response, encapsulated by a fibro-inflammatory barrier. On the other hand, PCL provoked a controlled inflammatory reaction and facilitated cell migration into the scaffold, although it induced a fibrotic response around PCL fibers. Conversely, the PEGDA-GelMA composite emerged as a promising candidate for intracerebral implantation. It facilitated the creation of a permissive glial layer, while also inducing neovascularization and attracting neuronal progenitors.

**Conclusion:**

Behavior, MRI monitoring and histology allowed a thorough following of biomaterial biocompatibility. The collective findings position PEGDA-GelMA as a convincing biomaterial option as a basis for treating severe brain lesions, offering new avenues in the search for effective treatments.

## Highlights

• A complex structure was printed close to the cerebral cortex organization in layers.
• High-resolution T2 MRI imaging effectively captured complex scaffold structures.
• Non-invasive MRI quantification of cerebral blow flow was achieved.
• It may be useful to monitor the progressive revascularization of implants.

## Graphical abstract

**Figure:**
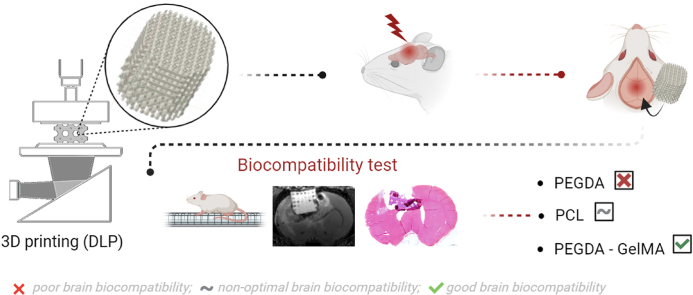

## 1 Introduction

Brain injuries such as stroke and severe head trauma are a global clinical concern due to their very high incidence in the adult population [1,2]. Today, acute brain injury is the leading cause of acquired disability in adults, with sensorimotor deficits sometimes persisting indefinitely after the traumatic event. Although cerebral plasticity can initiate regenerative processes after an accident, it is not sufficient to compensate massive neuronal loss. Even if stem cells or neural progenitors migrate to the core of the lesion, their survival remains very low. Furthermore, the vascular damage associated with injury, notably hypoxia, hemorrhage and edema, accelerates the necrosis of damaged neural tissue, creating an unfavorable environment for axonal regrowth. Alteration of the blood-brain barrier promotes the infiltration of blood-derived monocytes/macrophages which, together with activated microglia, extend inflammation beyond the initial lesion site, contributing to the expansion of secondary lesions [3,4]. Despite significant progress in understanding the pathophysiology of brain injuries, few therapeutic interventions have been validated in recent years. To overcome these obstacles, implantable biomaterials within the lesion have recently aroused interest in the treatment of brain lesions.

The development of biomaterial engineering has provided a new strategy for repairing damaged tissue. The core of tissue engineering is the construction of new tissue substitutes composed of synthetic or biological materials, with or without cells, to promote recovery and restoration of loss functions [5]. Biomaterials provide mechanical support and a flexible, controllable three-dimensional space. They replace the extracellular matrix for cell adhesion, growth and migration [6]. Implantable biomaterials must meet several criteria to guarantee safety and non-toxicity. Advanced biomimetic materials can also provide in situ guidance during axonal regeneration. Their architectures can guide axonal growth toward their biological targets, re-establishing efficient and even long-distance connections between damaged and healthy tissue [7]. These implants are manufactured using various techniques, including molding, electrospinning and self-assembly. However, the method offering the most control over the architecture and precision remains light-based three-dimensional (3D) printing. With recent developments in this technology, it has become possible to create reproducible scaffolds according to a pre-designed structure using computer-aided design (CAD) and to obtain more precise control over the macroscopic structure of the scaffold [8]. They serve as models for tissue regeneration and drive/guide new tissue growth. This enables the accurate reproduction of structures adapted to the complex physiology of tissues. Digital Light Processing 3D printing requires a biomaterial that is photopolymerizable and compatible with both additive manufacturing methods and biological systems, such as the brain. These limitations considerably reduce the range of useable materials [9]. Current studies on new biomaterials are focusing on biodegradable implantable scaffolds [[10], [11], [12]]. The idea behind developing degradable biomaterials is to optimize cell replacement and recolonization without leaving foreign materials permanently. Rigidity, biocompatibility, cell adhesion and bioresorbability are important parameters to consider. To date, few studies have investigated the in vivo reaction of biomaterials for brain reconstruction.

The objective of this study was first to design a scaffold compatible to cerebral cortex reconstruction and then to develop and test the biocompatibility of three candidate biomaterials that are both 3D printable and biodegradable or bioeliminable [[10], [11], [12]]. As no optimal slowly degradable biomaterial has yet been found for brain regeneration, the present study aimed to compare different biomaterials. Although highly informative, there are still very few comparative studies of various biomaterials [13]. We selected slowly bioresorbable materials (>3 months) capable of guiding axons in preferential directions and over distances compatible with the size of large brain lesions. Since various biomaterials, alone or in combination, have been suggested to ensure central nervous system regeneration [[13], [14], [15], [16]], we chose to compare the behavior of Poly (ethylene glycol) diacrylate (PEGDA), Polycaprolactone (PCL) and Poly (ethylene glycol) diacrylate (PEGDA) mixed with methacrylate gelatin (GelMA) in vivo in the brain-injured rat. PCL, and PEGDA-GelMA were chosen because had been found biocompatible with central nervous system studies [13,14]. PEGDA is a porous, bioeliminable hydrogel, particularly well-suited to photoreticulation printing. Its non-cytotoxicity has been tested in vitro on various cell strains and in vivo subcutaneously [17] or in several organs except the brain. That is why we wanted to test it in the lesioned brain. More specifically, the brain reaction to a PEG derivative appeared to be weak [18]. PCL is a semi-crystalline polyester particularly appreciated for its mechanical strength, biocompatibility and biodegradability [19]. Its slow degradation ensures long-lasting use, particularly for subcutaneous and surgical implants [20]. PCL's lack of cytotoxicity has been tested on several neural cell strains and in vivo in animal brains [21]. The material appears to be well tolerated, with generally a thin layer of fibrosis at the tissue-material interface [22]. GelMA, like PEGDA, gives hydrogels. Of natural origin, GelMA promotes cell adhesion thanks to its RGD sequences [23]. However, its rapid degradation rate and low mechanical strength limit its use on its own for fine structures below 300 μm, hence the interest in combining it with PEGDA to improve its mechanical properties while retaining its bioactivity [24].

This methodological study assessed the effects of these cerebral implants on sensorimotor recovery and tissue regeneration over several weeks. Technological approaches have been developed to provide a scaffold with a pattern, spatial resolution and porosity adapted to brain reconstruction, to ensure non-invasively MRI monitoring and, in the end, to establish a match with a detailed histological study.

## 2 Material and methods

### 2.1 Preparation of the implants

#### 2.1.1 Design for hydrogels

**Fig. 1:**
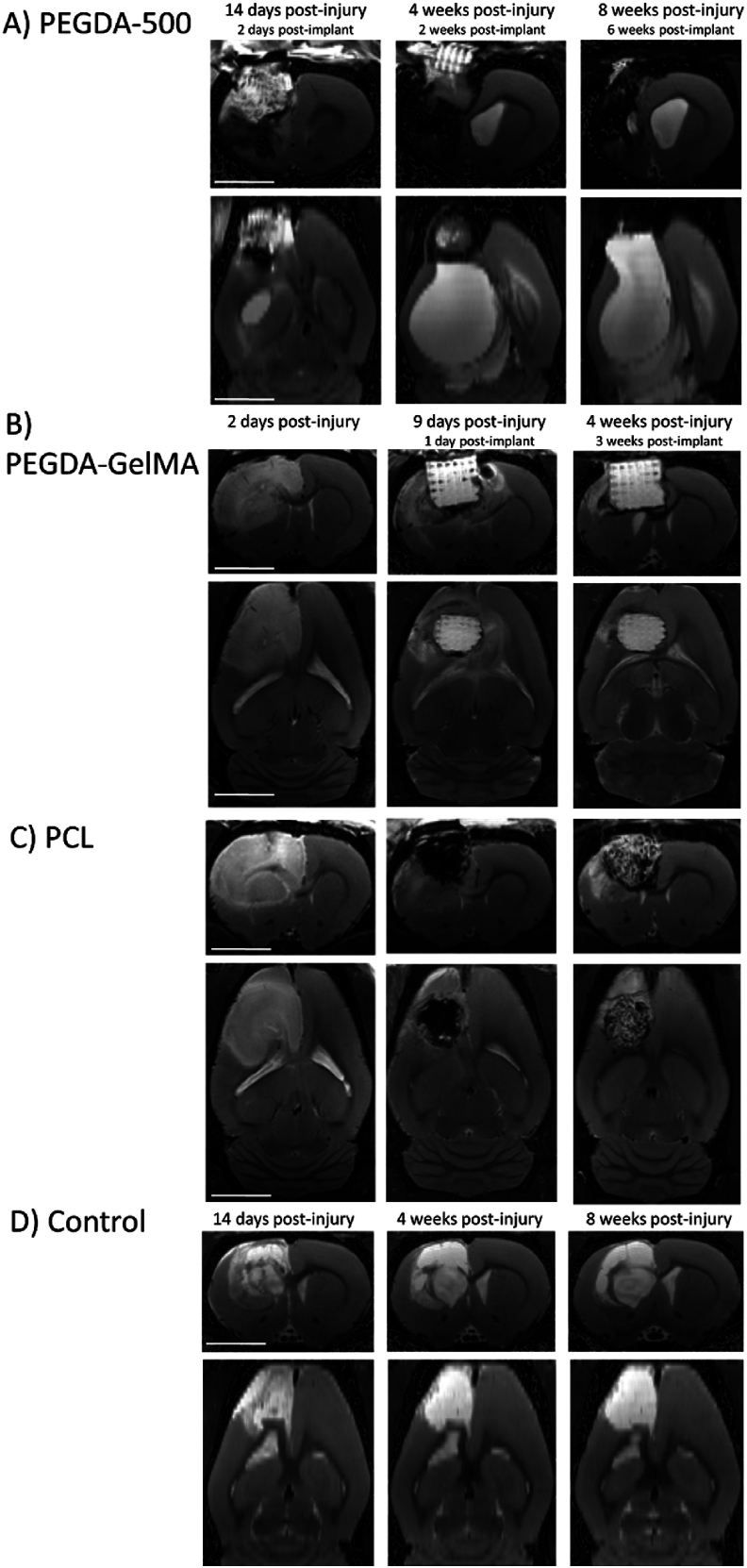
**Longitudinal MRI follow-up before and after implantation of a scaffold in a brain lesion.** Coronal and axial T2 MR images of the lesion and the implants. These implants were made with different biomaterials: PEGDA-500 (A), PEGDA-GelMA (B), PCL (C). A rat served as a brain lesioned control (D). PEGDA-500 was progressively rejected. PEDGA-GelMA and PCL were well tolerated. PEGDA-GelMA hydrogel was hyperintense on T2 images with no observable swelling. PCL was hypointense on T2 images and became progressively invaded with hyperintense cerebrospinal fluid. The lesion of the control rat went hyperintense with time because of necrosis and replacement by cerebrospinal fluid. Scale bars: 5 mm.

Post-injury MRI images (Fig. 1) were used to measure the lesion volume, and the volume of the implants was adapted to the lesion volume. A maximum implant height of 5 mm has been inserted. Moreover, the implants had to pass through the 5 mm diameter hole drilled in the skull to be inserted into the volume of the lesion. Implants were designed with Autodesk Fusion 360®, a computer-assisted design software. Structurally, the objective was to maximize porosity, cell infiltration and axonal guidance. The geometric fidelity, structural thickness and 1D swelling of the hydrogels in water at 25 °C were assessed by microscopic observation. The swelling was quantified by measuring the outer dimensions of the printed pieces which were compared with the dimensions of the CAD model (Suppl Fig. 1). Before printing the final implants, the difference between the 3D model of a prototype and what was actually printed was measured (Suppl Fig. 1B). The designs were optimized for the observation of transparent hydrogels with a simple optical microscope: non-symmetrical shape to be correctly oriented, repetition of the same patterns, a thick structure, no overlap of different patterns in the observation axis (Suppl Fig. 1. A).

**Fig. 2:**
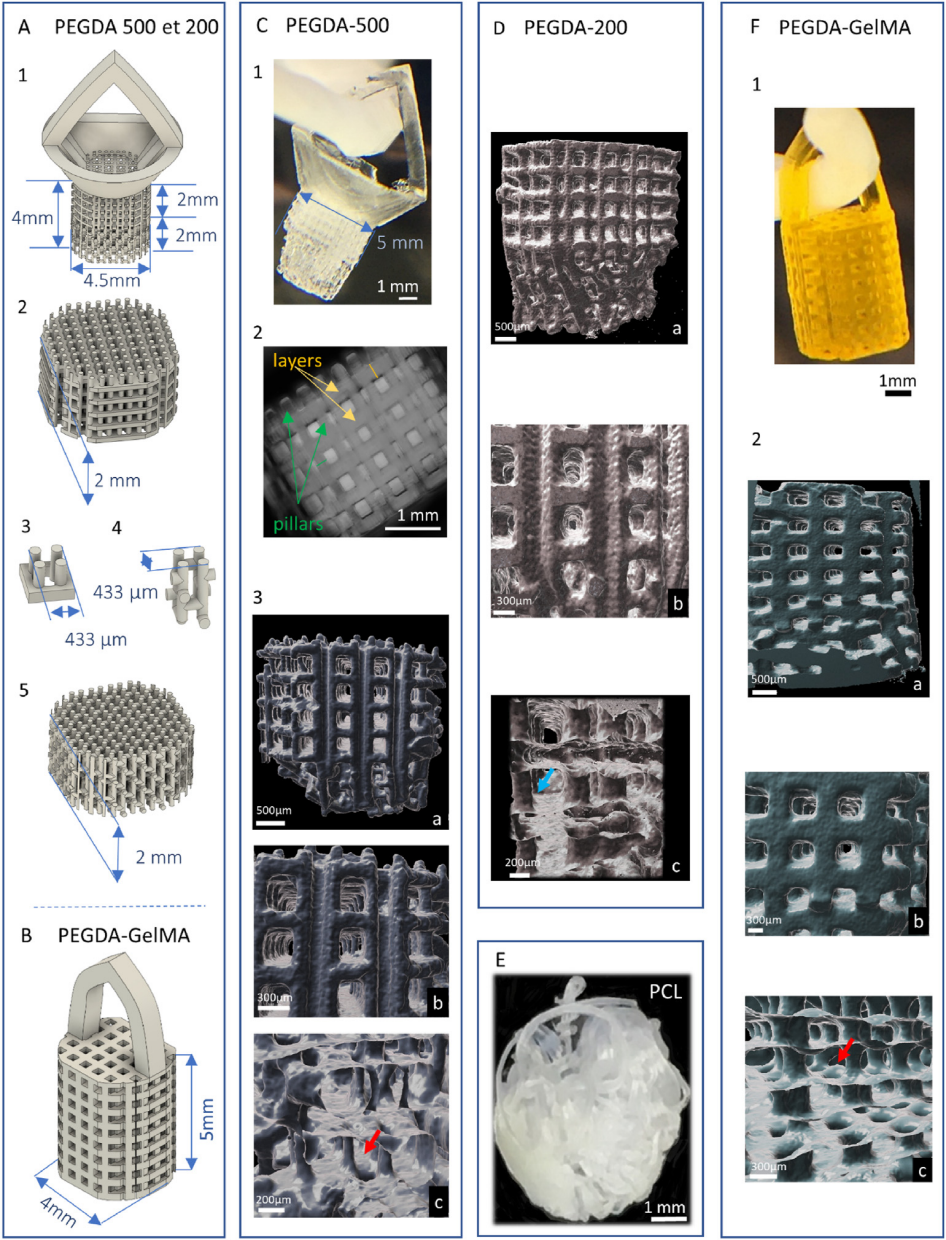
**Implant design and fabrication process**. (A) Detail of the complex PEGDA-500 and PEGDA-200 structures that are implanted. 1: CAD design used for PEGDA-500 and PEGDA-200. We can see on top an ogive-shaped handle used to manipulate the implants without damaging them. Below sits a funnel where cells can be placed (which was not used in the present study). The funnel and handle were cut off after implantation, so that only the lower sections remained inside the brain. 2: upper part with horizontal layers to support grey matter development. 3: Elementary pattern that makes up this upper part 4: elementary pattern that makes up the lower part. 5: lower part with mainly vertical pillars to guide axonal growth. (B) CAD design used for PEGDA-GelMA implants. A simpler design with a handle and a porous structure was printed with three privileged orthogonal directions. (C) 1: An example of a relatively fragile printed PEGDA -00 structure after the yellow photo-absorber was washed away. The width of the bottom part is 5 mm. 2: Microscopic view of the structure before being implanted. We can distinguish the pillars in one direction (green arrows) and, perpendicularly, the thicker layers (yellow arrows). 3: Micro-CT imaging of PEGDA-500 implant. Horizontal layers are perforated. The vertical pillars allow cells to pass through holes in the implant (red arrow). (D) Micro-CT imaging of PEGDA-200 implant. Guiding pillar from the top to the bottom of the construct (blue arrow). (E) The PCL implant consists of a thin PCL thread coiled into a ball about 5 mm wide. (F): 1: Printed structure of PEGDA-GelMA implant before washing the photo-absorber (width: 5 mm). 2: Micro-CT imaging. Horizontal layers are perforated. The vertical pillars allow cells to pass through holes in the implant (red arrow). a: Overview, b: Focus on architecture, c: Clipping plane, blue arrow: guiding pillar, red arrow: hole. X-ray microtomography was carried out at the Institut de Mécanique des Fluides de Toulouse (IMFT).

The PEGDA design (Fig. 2. A.1; Patent: PCT/EP2024/073072) consisted of three different parts: on top, a handle used to pick up the scaffold, then horizontal layers of material being configured to a cell growth radially along each layer (Fig. 2. A.2). Each layer comprises a plurality of openings for porosity and access to vertical pillars, and lastly, the vertical pillars to guide axonal growth. A remarkable feature is the presence of pillars guiding and traversing the structure from top to bottom. They enable a cell on the top layer to cross the structure in a straight line from top to bottom, or vice versa from bottom to top. They also enable cells on any layer to turn and grow on an intermediate layer. Only the last two parts remained in the brain. The horizontal layers of material served as scaffolds to guide the cellular organization in order to get closer to the cerebral cortex organization and were 2 mm high (Fig. 2. A.2). The vertical pillars below served as guides to regenerate the former axon bundles and were also 2 mm high (Fig. 2. A.5). We added anti-buckling horizontal beams to the pillars to prevent the implant from collapsing under its own weight or during implantation. These beams have been positioned according to a specific scheme that maximizes rigidity while minimizing the number of beams (Suppl Fig. 1. D-F). Pores width was determined based on previous experiments demonstrating optimal scaffold colonization by neural cells with channel widths of at least 60 μm [25]. The thickness of the structures could not be reduced any further due to the resolution of the printer and mechanical constraints.

Due to time constraints, the PEGDA-GelMA design was simpler and was not optimized for spatial resolution. It consisted of a highly porous scaffold with three privileged orthogonal directions (Fig. 2 B).

#### 2.1.2 Preparation of the PEGDA implants

We tested two types of Poly(ethylene glycol) diacrylate (PEGDA): PEGDA-200 Photo Ink™ and PEGDA-500 Photo Ink™ (CellInk Inc., USA). PEGDA is a bio eliminable and transparent hydrogel that can be photo-crosslinked. PEGDA-200 has a molecular weight greater than 2000 Da and is called “200” by CellInk because the storage modulus at the end of exposure is 200 ± 20 kPa. PEGDA-500 is composed of both low (<2000 Da) and high (>2000 Da) molecular weight components. It is stiffer, less transparent and its storage modulus at the end of exposure is 500 ± 25 kPa. Moduli are provided by CellInk and are measured by photorheology, using the following parameters: 100 μm gap, and 90s of exposition at 20 mW/cm^2^. The implants were printed with a LumenX™ (CellInk Inc.). This printer uses digital light processing (DLP) technology, with a wavelength of 405 nm (violet) and a horizontal resolution of 50 μm (x, y). We used the recommended printing parameters, i.e., a 50 μm layer height and a light power of 20 mW/cm^2^. For PEGDA-200 and PEGDA-500, the exposure times for the first layers were set at 22 and 9 s to ensure adhesion to the plate and subsequent layers had exposure times of 5.5 and 3 s, respectively. The implants in a bath of sterile Dulbecco's phosphate-buffered saline (PBS) and antibiotics (Penicilline-Streptomycine 1 %) were placed on an orbital shaker for a minimum of 24 h, during which time the bath was changed three times, in particular to evacuate residues of photocrosslinking agent, photoabsorber and prepolymer present in the printing ink. The implants were then stored in a PBS solution in the refrigerator at 4 °C. Finally, the biomaterials were functionalized by the addition of a Poly-d-Lysine (100 μg/ml; Sigma-Aldrich, during 5 h at room temperature), rinsed for 10 min 3 times with PBS (phosphate-buffered saline) and coated with laminin (40 μg/ml; Sigma-Aldrich) during 24 h at room temperature.

#### 2.1.3 Preparation of PEGDA-GelMA implants

PEGDA-200 PhotoInk with GelMA PhotoInk™ (CellInk Inc.) were combined in equal proportions. PEGDA-200 was chosen rather than PEGDA-500 because fewer stiff materials are thought to be more suited for brain compatibility. GelMA stands for Gelatin Methacrylate, a gel strength 300 (Bloom test), Type A gelatin that can be photoreticulated. Its storage modulus at the end of exposure is around 22 ± 5 kPa (provided by CellInk, photorheology, with following parameters: 100 μm gap, 90 s exposure at 20mW/cm2). Both hydrogels were separately warmed in water at 37 °C before mixing. We used the same printer (LumenX, from CellInk Inc.), setting a layer thickness of 50 μm, an exposure time for the first layers of 15 s, an exposure time of 5 s, and a light power of 20 mW/cm^2^. Precautions must be taken to prevent the gelatin from setting during printing. We encountered difficulties in assessing the exact proportion of PEGDA and GelMA in the mixture because GelMA partly jellified during the printing process. The implants were washed three times for a minimum of 24 h in PBS and Penicilline-Streptomycine 1 %.

#### 2.1.4 Preparation of PCL implant

We used Polycaprolactone (PCL) pellets from CellInk Inc., which have a molecular weight of 50 000 Da. The 100–500 μm filaments have a modulus of around 175–225 kPa. Due to the BioX (CellInk Inc.) fused deposition modelling (FDM) printer failure, we could not print a 3D scaffold. Instead, using the BioX printer, we extruded a thread of PCL and stretched it until it reached its tensile limit without breaking. The stretched thread was then wound to make a sphere. The end was heated to 70 °C and welded to the ball. It resulted in a soft and highly 3D porous structure (Fig. 2 E). The implants were washed three times for a minimum of 24 h in PBS and Penicilline-Streptomycine 1 %.

#### 2.1.5 Implant sterilization

The day before implantation, the implants were sterilized with 70 % ethanol filtered at 0.22 μm for 2 h in a multi-well plate. After ethanol treatment, the implants were washed three times for 10 min each with sterile PBS + Penicilline-Streptomycine (1 %). Sterilized implants were stored overnight in the refrigerator at 4 °C. On the day of implantation, the implants were exposed to UV light for 30 min and stored in a closed environment with PBS + Penicilline-Streptomycine (1 %) medium. All sterilization procedures, including the ethanol treatment, PBS washes, and UV exposure, were carried out under a microbiological safety station (MSS) in a level 2 safety laboratory.

#### 2.1.6 Handling of hydrogel

As no dedicated handling tools were available, nor described in the literature for fragile hydrogels, we fabricated tools using 3D printing or by shaping them from PTFE (polytetrafluoroethylene) rods, which were then autoclaved. We made a tool with a thin, narrow blade-like shape to remove the fragile structure from the LumenX build plate. Due to the low elongation at break, the hydrogels tended to break after repeated use. To avoid direct handling, we added a handle on top of the implants to pick them up with a hook (Fig. 2 A, B, C, F). The handle has an ogive shape. The tip was positioned vertically to the center of gravity so that the implant naturally stands vertically. The handle was removed during surgery.

#### 2.1.7 Porosity estimation

Porosity was calculated based on Eq. (1) from Ref. [26]:

(1) $$Porosity=1-\frac{V_{solid}}{V_{total}}\times 100\%$$

where V_solid_ = volume of solid, and V_total_ = total volume of scaffold.

For the simple shape of the PEGDA-GelMA structures, which is a pattern repeated in all three dimensions, we measured the pattern dimensions and calculated analytically its porosity to approximate that of the complete structure (Suppl Fig. 2).

For the complex shape of the PEGDA structures, the structures dimensions were measured with a brightfield microscope, then a CAD replica of the printed structure was built, and V_solid_ and V_total_ were calculated by the CAD software.

For PCL, we measured the wire length l, its mean diameter d, and the diameter D of the resulting sphere. We then calculated the total volume of wire, the volume of the sphere, and porosity, as shown in Eq. (2) below.

(2) $$PCL_{porosity}=1-\frac{\frac{\pi }{4}d^{2}l}{\frac{4}{3\times 8}\pi D^{3}}\times 100\%$$

#### 2.1.8 μComputed tomography

An EasyTom XL tomograph (RX Solutions) was used for X-ray tomography. Acquisition of the X-ray radiographs is made using a 150 kV Hamamatsu X-ray source. Image acquisition is performed at a 55 kV voltage and a 90 μA amperage. The exposure time on the flat panel sensor is typically between 1 and 2 s for each radiograph (depending on the sample considered) and 1440 radiographs are acquired as the sample rotates through 360°. Reconstruction is carried out using the X-Act software (RX Solutions), leading to tomographic slices. The isotropic voxel size depends on the exact positioning of the sample with regards to the X-ray source output window and the flat panel. It is set to 5 μm in the present study.

The sample was taken from the buffer solution and gently pressed against a blotting paper to remove the solution trapped by capillary forces within the structure. Despite this, some capillary water bridges may remain inside the structure and can be visualized on tomographic sections (note that the contrast between the liquid bridges and the hydrogel is not enough to remove the liquid through image segmentation). The sample is place in a closed plastic vial, at the bottom of which some cotton wet with solution is placed, to obtain a wet atmosphere and limit the sample drying during the tomographic scan which last around 1 h. Images were processed with Fiji and Imaris.

### 2.2 Animals

Twenty-two female Sprague-Dawley rats (280–320 g, 11-weeks-old, Janvier, France) were housed two per cage (30 cm length, 18 cm height, 32 cm width), in a controlled environment (20 °C) under a 12 h/12 h light/dark cycle with free access to food and water. No gender difference has been observed in this brain lesion model [7,27]. Sham group*: n* = *8*; Injured group: *n* = *8*; PEGDA group: n = 2 (PEGDA-500 *n* = *1*; PEGDA-200 *n* = *1)*; PEGDA-GelMA group: *n* = *3*; PCL group: *n* = *1*. We chose these numbers to carry out case studies and assess whether each implanted rat fell within the variability of the group of control rats. For PCL, a single rat was tested in a pilot experiment. Animals were treated according to the Council of the European Communities guidelines (EU Directive 2010/63). The protocol was approved by the “Direction Départementale de la Protection des Populations de la Haute – Garonne” and the “Comité d’éthique pour l'expérimentation animale Midi- Pyrénées” (protocol n° APAFIS#22419-2019101115259327v5). According to 3R recommendations, this pilot study used the smallest number of animals necessary: in case of biomaterials rejection by the brain, two animals were considered sufficient to eliminate the biomaterial. Enrichment was available and all efforts were made to reduce suffering. All the experiments have been performed following the ARRIVE guidelines.

#### 2.2.1 Surgical procedures

**Fig. 3:**
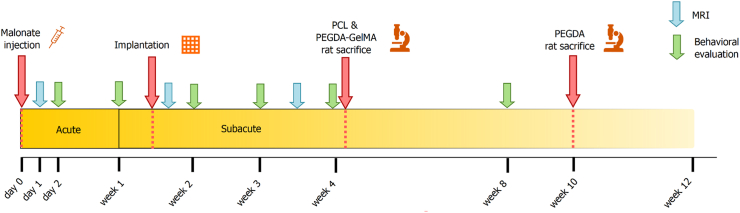
**Study protocol overview.** Red arrows (key experimental steps): malonate injection for lesion induction, scaffold implantation into the lesion, and animal sacrifice for further histological analysis of the cytoarchitecture and biocompatibility. Green arrows (behavioral tests): for training, tests started before injury and were then performed at different time points post-injury: one, two, three, four, and eight weeks. Blue arrows (MRI acquisitions): for PEGDA, post-implantation scans were carried out at one, 22, and 52 days; for PCL and PEGDA-GelMA at one day post-injury, one day post-implantation, and 17 days post-implantation. Yellow strip: represents the acute phase (first week) after the brain lesion, and then the subacute phase. PEGDA was implanted 13 days post-injury. PCL and PEGDA-GelMA were implanted eight days post-injury.

Rats were anesthetized with isoflurane (3 % for induction, 3–5% for maintenance, in 0.7 l/min O_2_), secured to a stereotaxic frame (Bioseb lab, France). Pre-medication with an intraperitoneal injection of methylprednisolone (20 mg/kg, Centravet, France) and a subcutaneous injection before the scalp incision of lidocaine 2 % (4 mg/kg, Centravet) were used for analgesia. Body temperature, measured by a rectal probe, was maintained at 37 °C using a homeothermic blanket. Cortical lesion of the motor area (M1) was induced by malonate injection (5 μL, 3 M solution, pH 7.4 in phosphate-buffered saline (PBS); Sigma-Aldrich, France) in *n* = 14 rats at the following stereotaxic coordinates: 2.5 mm lateral and 0.5 mm ahead to Bregma, with 2 mm depth [28]. PBS was used for the sham group (*n* = 8). The lesioned hemisphere was the dominant one, identified through the grip strength test (see below). This model was previously validated and published by our group [27,28]. Animals were operated on again 8–13 days later for implantation, during the peak of neurogenesis, using the same procedure (Fig. 3). To introduce the implants into the lesion, a 5 mm diameter cranial flap was drilled, removed and put back afterwards. The implants have a stiffer structure compared to the brain (which has a shear modulus in the range of 0.4–1.4 kPa [29]) and thus were easily inserted. A detailed protocol of the study is available in Fig. 3.

#### 2.2.2 Motor tests

The rats’ behavior was monitored throughout the study to follow the development of clinical signs and potential effects of the biomaterial.

#### 2.2.3 Grip strength test

The grip strength test measures the maximal forelimb muscle grip strength. Rats were trained for 2 weeks before the surgery. A dynamometer measured the maximum isometric force (in Newton). This test was used to identify the dominant paw of the rat. It was validated in previous studies of our group [27,28,30,31]. Measures were made (three trials/paw/day) on two consecutive days, and a mean score was calculated for each week, before and after the injury. Values represent the strength of the front paw contralateral to the injected hemisphere compared to the pre-injury value, expressed as percent.

#### 2.2.4 Neurological severity scale (NSS)

Sensorimotor function was evaluated by the NSS which includes five tests based on reflexes, stability, sensitivity and depression. Motor ability was tested by hanging the animal 2 cm above a bench, while being suspended by the tail, to determine if the rat was able to touch the bench top with its paws and move forward (healthy reaction). Beam balance was used to assess the rat's motor and sensitive abilities. The evaluation of proprioception consisted in putting the impaired forelimb and hindlimb to the edge of a bench, to assess retraction. Finally, grooming behavior was observed since a lack of grooming in rats can reflect “pseudo-depression” [32]. All tests were scored on a scale from zero to 16 points. The higher the score, the more severe the deficits [28].

### 2.3 MRI

Imaging was performed on a 7T preclinical MRI scanner (Biospec 70/16, Bruker, Ettlingen, Germany) equipped with a volume transmit coil and a 2 × 2 elements surface receiver coil. Within the magnet, animals were placed in a thermo-regulated imaging cell to ensure a 37 °C body temperature. Anesthesia was maintained by inhalation of an Isoflurane/O2 mixture set between 1.5 % and 2.5 % depending on the animal respiratory rate.

The following acquisitions have been conducted. Firstly, anatomical imaging in T2 weighting using a T2 Turbo-RARE sequence with TE/TR = 35.7, 5452 ms and coronal slices with a spatial resolution of 0.137 × 0.137 × 0.500 mm^3^. The acquisition time was 17 min 27 s. Axial slices with a spatial resolution of 0.137 × 0.137 × 0.500 mm^3^ were also recorded for the PEGDA-GelMA and PCL rats.

Secondly, perfusion imaging: cerebral blood flow maps were acquired using pseudo-continuous arterial spin labelling combined with an inversion efficiency measure, consistently with previous studies [33]. The pCASL-EPI sequence was acquired as follows: TE/TR = 15/5000 ms, coronal slices with 0.326 × 0.326 × 0.800 mm^3^ spatial resolution, 50 repetitions, a labelling time (LT) of 4s and a post labelling time (PLD) of 300 ms. The acquisition time was 8 min 20 s. The T1 map was acquired with a FAIR-MTI sequence with EPI readout (same parameters as of the pCASL sequence) with a TR of 10 s and 17 inversions-recovery between 20 and 9000 ms of TI. The acquisition time was 2 min 50 s. Perfusion maps were estimated using MP3 software, considering the single compartment model and an arterial transit time (ATT) equal to 300 ms [34,35]. Perfusion image processing is described more extensively in Labriji et al. [36].

Finally, diffusion imaging consisted of DTI-EPI sequences with the following parameters: TE/TR = 33.3/6500 ms, 5 vol at b = 0; 30 diffusion directions at b = 700 s/mm^2^, 5 averages, and coronal slices with a spatial resolution of 0.176 × 0.176 × 0.800 mm^3^. The acquisition time was 18 min 57 s. The fractional anisotropy (FA), mean (MD) and radial diffusivity (RD) maps were computed with the FSL DTIFIT tool.

### 2.4 Histology

At the end of the study, rats (all implanted rats, and one rat in the sham group) were anesthetized with isoflurane (3 % for induction) and then rapidly received 0.3 ml of heparin and a lethal injection of 1 ml of sodium pentobarbital (159 mg/kg, Centravet) intraperitoneally. Intracardiac perfusion of heparinized% NaCl (200 ml, 20 min) was used to remove blood from the vessels and was followed by a 4 % paraformaldehyde (PFA) perfusion (250–300 ml, 40 min) for tissue fixation. Brains were then extracted and immersed in a 10 % buffered formalin bath for 24 h of post-fixation at 4 °C. They were subsequently embedded in paraffin using an embedding device (Leica EG 1160) at the histo-pathology platform of the ENVT (Ecole Nationale Vétérinaire de Toulouse, LabHPEC). The cold “brain + paraffin” block was sectioned with a microtome (Leica SM 2010R). For each brain, about 100 coronal sections of 8  μm thickness were made. One out of twelve sections were stained with Hematoxylin Eosin (HE) and Cresyl Violet acetate, according to standard procedures (Hematoxylin Eosin stain and Nissl stain). The HE-stained slides were examined under a white light microscope and digitized with a digital slide scanner (Panoramic desk 3D HISTEC). These images were compared to the pre-sacrifice MRI images. Brain sections at the level of the lesion underwent immunohistochemical staining with glial fibrillary acidic protein (GFAP), astrocytic marker, mouse monoclonal antibody, clone 6F12 Dako, low pH (citrate pH 6) retrieval antigen, 1:50 dilution. They were also stained for GRAM (marker of the bacterial wall) and Masson's Trichrome (collagen fiber marker).

For immunofluorescence staining, the section levels were identified using the Paxinos and Watson atlas [37]. Immunofluorescence on paraffin-embedded sections requires prior unmasking of the sections in Tris EDTA buffer (pH 9) in the microwave at 300W for 10 min. The immunolabelling was performed directly on the slides using a PAP Pen. The labelled slides were placed in a humid chamber at room temperature and incubated for 30 min with a blocking buffer of 1 % bovine serum albumin (BSA, Sigma-Aldrich) and 10 % serum (donkey or goat, thermo Fisher Scientific or Sigma-Aldrich, respectively) to block non-specific sites. The sections were then exposed with primary antibodies for 90 min, using the appropriate dilution: rabbit anti-NeuN 1:3000 (Abcam#ab177487); goat anti-DCX 1:200 (SantaCruz#sc-8066); rabbit anti-Tuj1 1:500 (neuron-specific class III beta-tubulin; Covance#PRB435P); Lectin from Lycopersicon esculentum (tomato) monoclonal conjugate with FITC 1:200 (Sigma-Aldrich #L0401). After three 10-min washes at room temperature with washing buffer (PBS 1X + 0.5 % BSA), the sections were incubated with the specific secondary antibodies coupled to a fluorochrome, for 50 min at room temperature in the dark. After 3 further 10-min washes at room temperature with washing buffer, fluorescent mounting medium (Fluoroshield™ with DAPI, Sigma-aldrich) was used to mount the sections and counterstain the nuclei with incorporated 4′,6-diamidino-2-phenylindole (DAPI) at the same time. Images were captured using a Nikon Eclipse Ti2 series fluorescence microscope and analyzed using Fiji and Imaris image analysis softwares.

### 2.5 Statistical analysis

The GraphPad Prism 9.41 software was used for analyses. Data are presented as median and interquartile range. Wilcoxon tests were used to compare different time points in each experimental group. The Pearson test was used for correlation with a 95 % confidence interval.

## 3 Results

### 3.1 Scaffold printing and implantation procedure

A scaffold with a complex shape was printed with patterns, spatial resolution and porosity adapted to neural reconstruction (Fig. 2C, D). An attempt to mimic the brain was achieved: the top part of the implant was designed to reconstruct cortical layers whereas the bottom part served as axon guides to reform white matter bundles. Fig. 2C3, D, and F.2 show μCT images of the hydrogels revealing the overall porous architecture, the organization in interconnected layers and pillars, and the interior of the constructs. Using an optical microscope, we measured a 1D swelling in water of 30 % for PEGDA-200 (Suppl Fig. 1.B) and 10 % for PEGDA-500 (Fig. 2C). Layer thickness was respectively 210 μm and 250 μm with pillars widths of 150 μm and 160 μm, and distances between consecutive layers of 360 μm and 250 μm. Based on these measurements, we estimated a 3D porosity of 67 % and 54 % respectively. The PEGDA implant width was 5 mm before implantation (microscopic observation) and remained 5 mm after implantation according to MRI data (Fig. 1). The PEGDA-GelMA implants displayed a 1D swelling of 12 % in water (Fig. 2F). Holes widths range from 240 to 310 μm, and pillar widths from 344 to 420 μm. The estimated porosity, based on those measurements, was 27 %. The implant had a width of 4.5 mm before implantation and remained 4.5 mm after implantation according to MRI data. The modulus of implants were respectively 560 ± 230 kPa, 240 ± 110 kPa and 90 ± 50 kPa for PEGDA-500, PEGDA-200 and PEGDA-GelMA implants (Suppl Fig. 3). Cutting off the extra parts of the implants during surgery (handle and funnel) was straightforward given the material softness. The PCL thread had a diameter of 50 μm ± 16 μm with a length of 2000 mm. The calculated porosity was 91 %. Using MRI data recorded one day after implantation, the PCL implant diameter was found to be between 4 and 5 mm.

### 3.2 In vivo biocompatibility of materials

#### 3.2.1 Grip strength test and neurological severity scale Neurological severity scale (NSS)

**Fig. 4:**
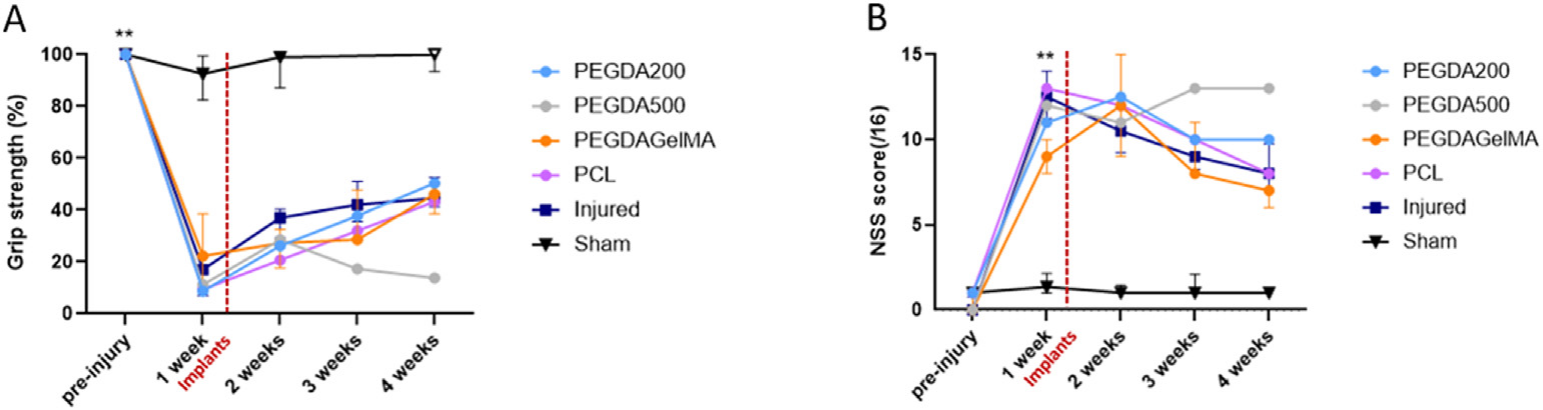
**Follow-up by behavioral tests following injury to the sensorimotor cortex and implantation of regenerative implants**. (A) Grip strength test shows the grip strength of the front paw contralateral to the injected hemisphere compared to the pre-injury value, expressed as %. The Wilcoxon test compares the values measured one week post-injury to the pre-injury ones for the injured (grey squares) groups (∗∗p < 0.01). (B) The NSS score shows the sensorimotor deficits after malonate injection (scored out of 16). Injured group is significantly impaired one week post-injury compared to pre-injury value (Wilcoxon test, ∗∗p < 0.01). The graphs show the median and the interquartile range. Sham group*: n* = *8*; Injured group: *n* = *8*; PCL group: *n* = *1*; PEGDA-GelMA group: *n* = *3*; PEGDA-500 group: *n* = *1*; PEGDA-200 group: *n* = *1*. PCL: polycaprolactone; PEGDA-GelMA: Poly(ethylene glycol) diacrylate Gelatin methacrylate; PEGDA: Poly(ethylene glycol) diacrylate.

The grip strength test measures the grip strength of the forepaw contralateral to the injected hemisphere expressed in percentage of the pre-injury value. Uninjured rats receiving PBS during surgery (sham) had constant grip strength (Fig. 4A). One week following malonate injection, injured rats show a significant reduction in the grip strength of their dominant paw to 16.0 % (p < 0.01, n = 8). They demonstrated a spontaneous recovery reaching 40 % by the end of one month. At one-week post-lesion, the deficit was identical for rats that would be implanted. Rats receiving PCL, PEGDA-GelMA and PEGDA-200 implants exhibited a grip strength recovery similar to the injured control group. Same results were obtained for the NSS that showed the sensorimotor deficits following malonate injection (score out of 16) (Fig. 4B). The injured group was significantly impaired with a score of 12.5 (p < 0.01, n = 8) one week after injury compared to the pre-injury value. They recovered at 9.0 at 4 weeks. The rat implanted with the PEGDA-500 scaffold had a secondary lesion at the time of implantation independent of the biomaterial but that impacted recovery. Its behavioral measures are therefore displayed in light grey line at three and four weeks. These behavior tests revealed no worsening of the deficits associated with the implants. No side effects were observed in rats implanted with PCL, PEGDA-GelMA and PEGDA.

#### 3.2.2 Visibility of biomaterial on MRI and follow-up

MRI T2 imaging was particularly effective for non-invasive follow-up of acceptability and degradation of the different biomaterials. Hydrogels appeared hyperintense on T2 images while PCL was dark (Fig. 1). The MRI 137 μm spatial resolution made it possible to image 150 μm structures, allowing easy identification of scaffold layers and pillars. PEGDA-500 was progressively rejected (Fig. 1). An abscess developed around the PEGDA-200 implant (Suppl Fig. 4). The implant, which appears hyperintense, is embedded in an intermediate-intensity matter, the whole being delimited from the parenchyma by a hypointense rim (Suppl Fig. 4.A). Subsequent histological examination and Gram staining confirmed a staphylococcus infection in this rat (Suppl Fig. 4.B3). The hypointense rim observed in the MRI corresponds to a fibrotic scar, as further validated by histological results (Suppl Fig. 4.C, D). Furthermore, fibroblastic hypercellularity is known to appear hypointense on T2 images [38]. Apart from the infection, PEGDA 200 displayed a typical foreign body reaction with macrophages encircling the biomaterial (Suppl Fig. 4.B3). PEDGA-GelMA and PCL did not present signs of rejection and seemed well tolerated. PEGDA-GelMA hydrogel was hyperintense on T2 images. For both PEGDA-200 and PEGDA-GelMA, in vivo MRI data revealed unchanged outer dimensions of the implants throughout in vivo testing, suggesting no further swelling and little or no degradation (Fig. 1). Due to minor hemorrhage from the surgical procedure, blood filled the pore spaces of the PCL scaffold, causing them to appear hypointense in the dark PCL the day after implantation (Fig. 1C middle). Three weeks after, the scaffold became progressively invaded with hyperintense cerebrospinal fluid (Fig. 1C right). The lesion of the control rat went hyperintense with time because of necrosis and replacement by cerebrospinal fluid (Fig. 1D).

### 3.3 Biomaterials that induced adverse tissue reaction

**Fig. 5:**
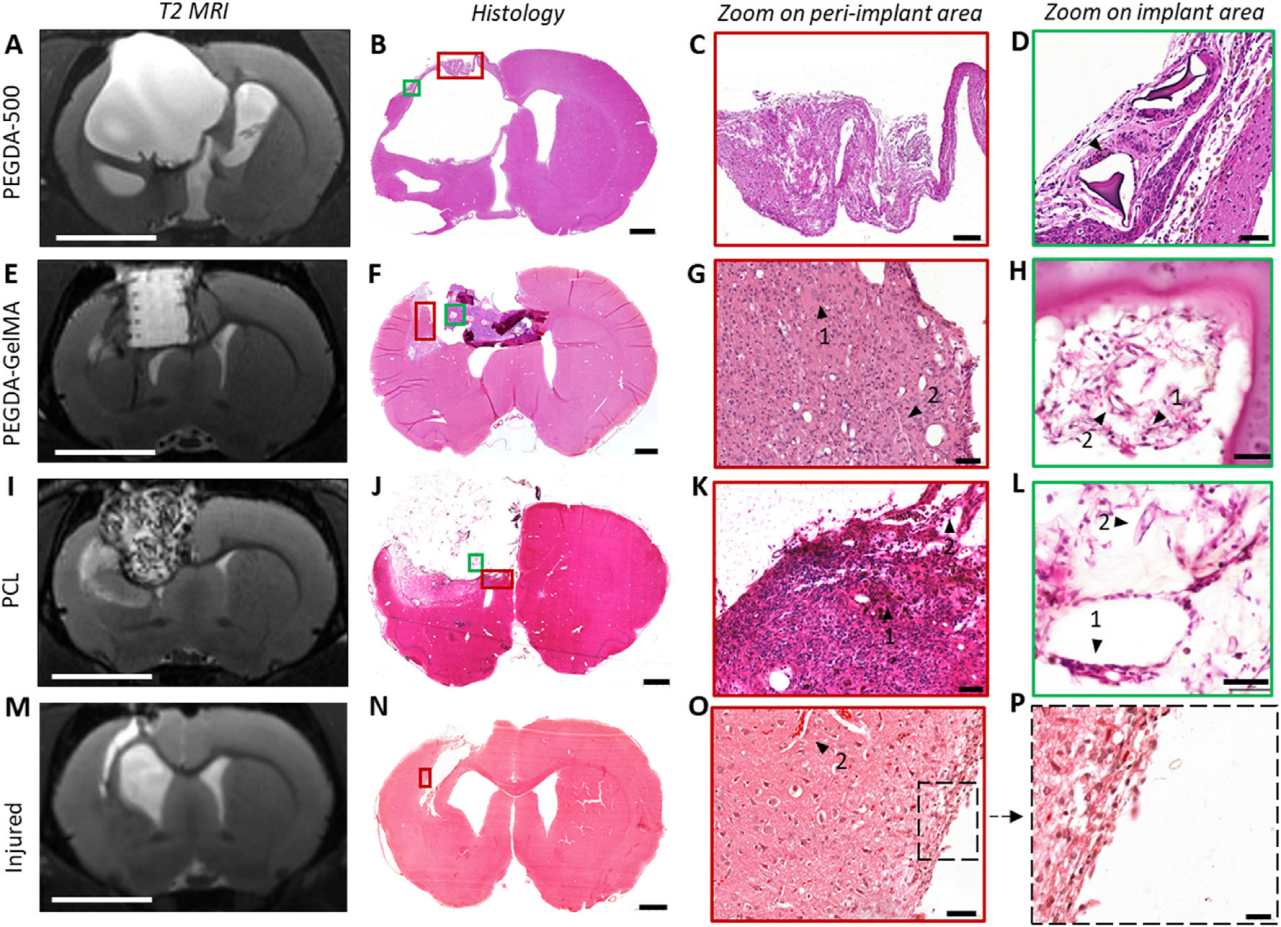
**T2 MRI and histological sections of control and implanted lesioned rat brains.** (A) Brain T2 MRI 2 months after a large lesion and 6 weeks after the implantation of PEGDA-500 showing ventricular dilation, section at bregma −0.60 mm. Scale bar: 5 mm. (B) HE brain section at 2 months, bregma −0.60 mm. Severe ventricular dilation associated with implant rejection. (C) Boxed area in B, red frame. Folded layer of brain tissue surrounding major ventricular dilation ipsilateral to the lesion. (D) Boxed area in B, green frame. Foreign body reaction around pieces of implant residue surrounded by epithelioid macrophages and multinucleated foreign body giant cells. Presence of surrounding hemosiderin pigments in macrophages associated with previous local hemorrhage. (E) T2 MRI 1 month after the lesion and 3 weeks after the implantation of PEGDA-GelMA, bregma 0.12 mm. Implant overlying the cortical-striatal lesion area, visualization of architectural patterns. (F) HE section of brain section 1 month after implantation showing the implant folded over the lesion site, bregma 1.56 mm. (G) Perilesional brain tissue showing chronic inflammatory reaction and granulation tissue including ^1^macrophages, fibroblasts and ^2^capillaries. (H) Cell migration and colonization of 3D patterns, creating a delicate, densely neovascularized fibroblastic tissue. Presence of haemosiderin^1^–laden macrophages and red blood cells^2^. (I) T2 MRI 1 month after the lesion and 3 weeks after the implantation of PCL, bregma 0.00 mm. Implant overlying the cortical-striatal lesion area, visualization of filaments of the 3D architecture. (J) 1-month PCL rat brain HE section, bregma 2.28 mm. Spherical lesion molded to the scalloped edges of the 3D implant. (K) Perilesional brain tissue showing a marked chronic inflammatory mononuclear reaction including ^1^macrophages, fibroblasts, ^2^neocapillaries and hemosiderin deposits. (L) High magnification of the delicate neoformed tissue inside the 3D porous implant showing the presence of fine collagen fibers, fibroblasts, macrophages^1^ and neovessels^2^. The foreign body reaction induced by the PCL biomaterial is identified by the presence of multi-nucleated foreign body giant cells. (M) T2 MRI 1 month after the lesion, bregma 0.24 mm. The ventricle is dilated due to atrophy of the striatum. (N) 1-month control rat HE brain section with empty space created by injury and slight dilation of the ventricle, bregma 0.96 mm. (S) The peri-injured brain tissue shows virtually no inflammatory reaction or neovascularization. Normal brain tissue can be seen all around the injured area. (O) Boxed area in S (zoom) showing no colonization of the lesioned area by cells. Scale bar: 1000  μm (left histology); 50  μm (for histology zoom middle and right) except for (P), 20  μm. HE: hematoxylin-eosin.

These biocompatibility experiments revealed that brain tissue does not well tolerate PEGDA. Indeed, biomaterial rejection was observed in the **PEGDA-500** rat. This rat showed histological rejection of the implant with highly inflammatory tissue reactions and expulsion of the implant outside the brain concomitant to major ipsilesional ventricular dilation (Fig. 1, Fig. 5D). Foreign-body reactions were present around macrophage-encapsulated implant pieces such as multinucleated giant cells (Fig. 5D). For the **PEGDA-200** rat, there was a granulomatous reaction, typical of biomaterial rejection (Supp Fig. 4B and C3).

Both PEGDA-200 and PEGDA-500 generated strong inflammatory reactions characteristic of foreign-body reactions that were independent of the infection for the PEGDA-200 rat. The tissue was highly inflammatory and therefore offered no possibility of tissue reconstruction or regeneration.

### 3.4 Biomaterials that were biocompatible

**Fig. 6:**
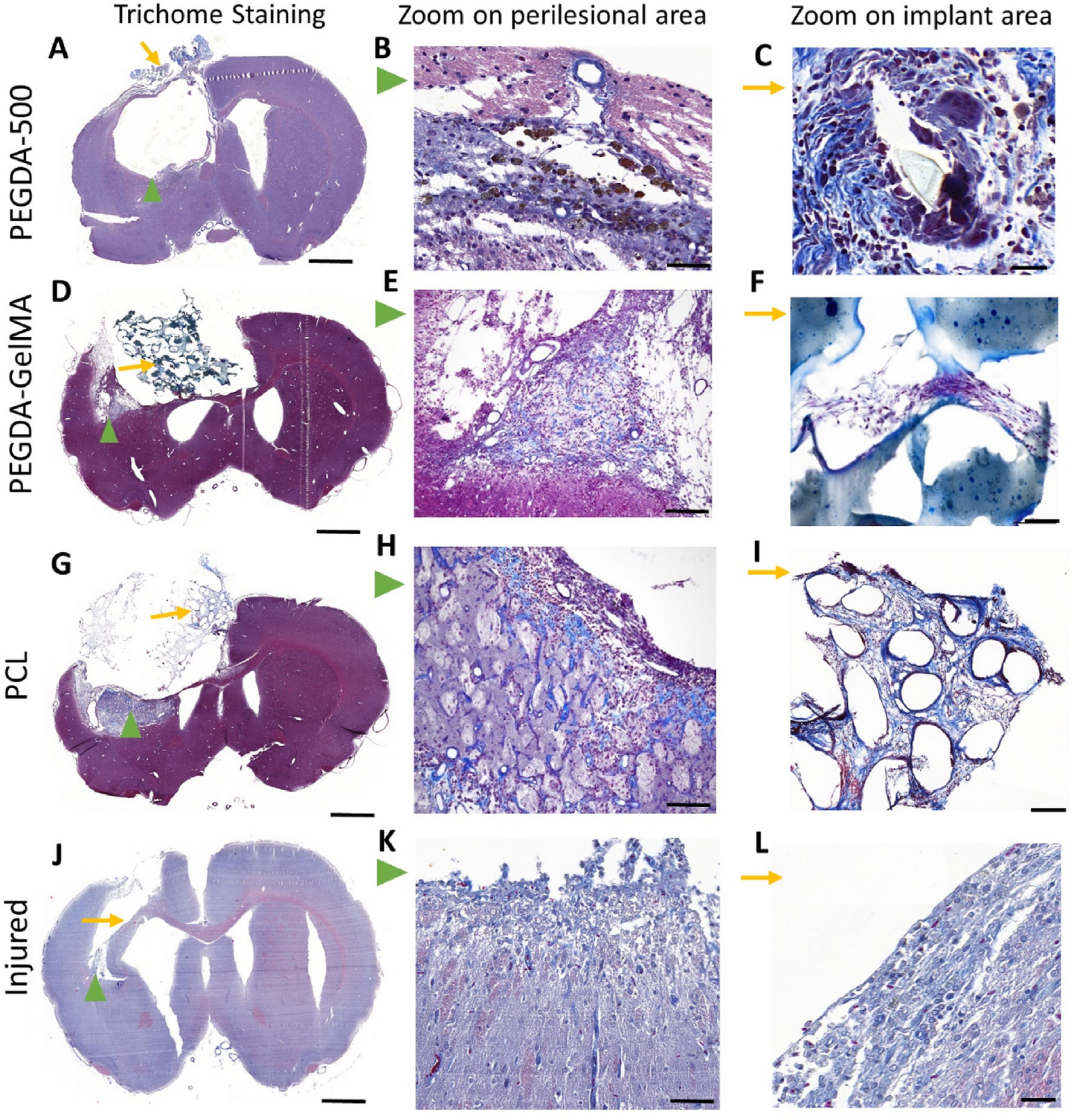
**Histology of trichrome stained brain sections and characterization of perilesional and implanted areas**. **(A, D, G, J):** topography of lesions at low magnification on coronal sections stained with Masson's trichrome *(left).* The collagen deposit is identified by the blue coloration surrounding the implants. Higher magnification shows perilesional brain tissue (green arrowhead). For PEGDA-500 **(B),** we can see many collagen fibers in the tissue and collagen rings around the vessels. For PEGDA-GelMA **(E),** the staining is weak and physiologic with few loose fibers in the tissue, and collagen rings around the vessels can be seen. For PCL **(H),** there are remnants of nerve fibers (purple) at the periphery of the implanted area with mature vessels (dark blue) and a tight collagenous stromal matrix is present. The non-implanted injured rat **(K)** shows an abrupt transition between healthy brain tissue and the lesion cavity, with few collagen fibers at the edge of the lesion and collagen around the vessels. **(L)** Edge of the lesion shows scarce collagenous fibers within the forming glial scar with some vessels surrounded by blue collagen. The peri-implant reactions are illustrated at higher magnification (C, F, I orange arrows). For PEGDA-500 **(C):** the residual implant material is surrounded by a macrophagic giant cell granulomatous reaction and associated with chronic blue collagen ring deposition. For PEGDA-GelMA **(F):** a fine, loose collagenous tissue colonized and filled the empty spaces in the biomaterial. Around PCL **(I)**, we observe the presence of fibroblastic cells and fibrovascular tissue with mature collagen fibers that are more packed. Scale bar: 1000 μm *(left);* 100 μm *(middle);* 50 μm *(right).*

In the present study, the assessment of biomaterial biocompatibility is mainly characterized by the absence of rejection or by the low extent of the inflammatory reaction (macrophages, fibrosis, glial scar) that could lead to further damage to the injured tissue. Of the three biomaterials tested in this study, only the PEGDA-GelMA and the PCL appeared to be biocompatible with nerve tissue. For the PEGDA-GelMA and PCL rats, GFAP staining showed a variability in the glial scar around the implant: absence of glial scar, a thin scar or a dense scar depending on the location. Of note, the PEGDA-GelMA and PCL rats showed larger lesions than the implanted area, which are described in Fig. 6 (D, E, G, H, green arrows) and 7 (A2, B2, A3, B3, green arrows) and are not directly related to the reaction to the biomaterial. These partially lesioned areas were made up of pre-existing brain parenchyma, which was slightly inflamed, vacuolated, and remodeled. They may correspond to a moderately damaged area after injection of malonate.

The **PEGDA-GelMA** rat had an implant in the center of the lesion that was connected to the surrounding tissue, which has geometrically structured lesion edges that follow the surface and pores of the implant (Fig. 5F). Note that the section shows a bent end of the biomaterial due to the histological technique (embedding, cutting and mounting of the brain sections on slides). The peri- and intra-implant area was made up of neoformed tissue with very dense oriented neovascularization. The neocapillaries were guided with chemotaxis, which attracted them towards the implant. Vascular structures with endothelial cells and pericytes appeared to come from the meningeal vessels. The tissue reaction was weak, with few loose collagen fibers in the neoformed zone (Fig. 6F). Cells had invaded and filled various implant gaps in the implant, moving from one gap to the next (Fig. 6F). Thin, delicate neoformed tissue was visible in the lacunae, with spindle-shaped stromal cells synthesizing loose collagen fibers, resembling the physiological extracellular matrix rather than a fibrous scar.

This biomaterial did not cause a major inflammatory response in contact with the tissue, as we have seen with the two previous biomaterials, PEGDA-200 and PEGDA-500. Instead, the host cells effectively colonized the gaps in the material.

In the **PCL** rat, although PCL is not visible on histology, the transparent spherical implant has left its imprint on the scalloped lesion edges (Fig. 5J). There was condensation and accumulation of mesh all around the thread holes dissolved by the histological technique. There was a granulomatous tissue reaction to the biomaterial with the presence of some multinucleated giant cells around the implant and a delineating fibrosis. Around the implant (Fig. 6I), the tissue was composed of spindle cells, corresponding to fibroblasts, and showed a denser collagen-associated framework than in the rat transplanted with PEGDA-GelMA (Fig. 6F).

Because these biomaterials are degradable or eliminable, some inflammatory response is expected. However, this reaction is not pro-inflammatory.

**Fig. 7:**
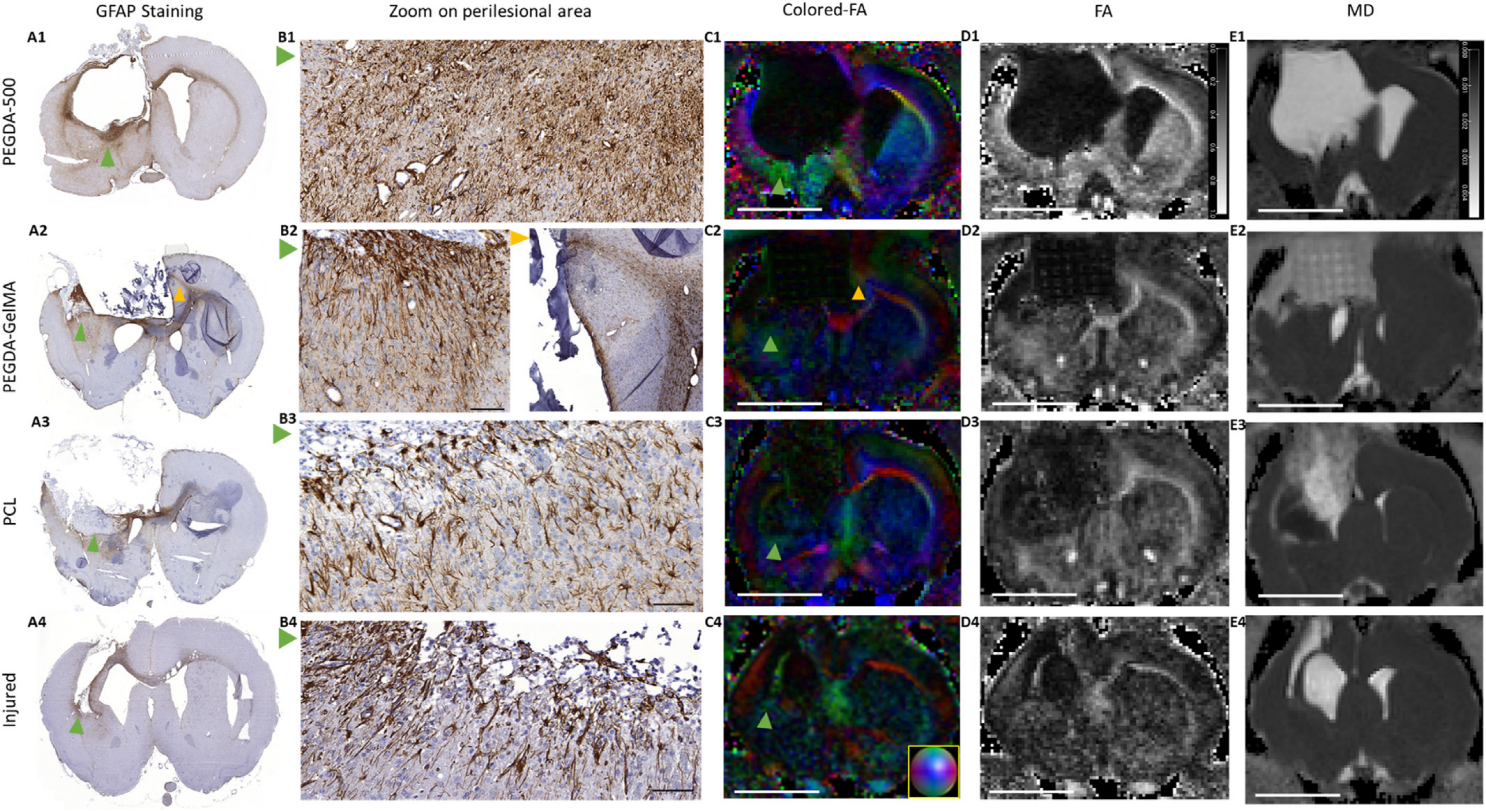
**Histology of brain sections and Colored-FA, FA, MD using MRI: characterization of the glial scar.** (**A1-A4**): Glial fibrillary acidic protein (GFAP) immunostaining was used to identify glial scar formation after lesion and implantation. Green arrows highlight (zoom **B1–B4**) the perilesional area with glial scar formation. Astrocytes can have different morphologies: there are thin aligned astrocytes perpendicular to the lesion with palisading effect (**B2, B3, B4**), others are hypertrophic with activated cytoplasm and extensions (**B1**). There are a few positive cells in the PEGDA-GelMA implant. Vascular overlay can be observed in all conditions. **(C)** MRI water diffusion images of coronal slices of rat brain. Colored-FA (fractional anisotropy) images show main directions of water diffusion (green: vertical; blue: antero-posterior; red: left-right). Main large bundles are visible: corpus callosum, anterior commissure. Lesion edges are also colored because of anisotropic water diffusion. The dense scar can be seen on MRI. **(D)** FA images show anisotropic water diffusion regardless of direction. **(E)** MD (mean diffusivity) quantifies water diffusion speed in mm^2^/sec. Water in ventricles is hyper-intense because of non-restricted diffusion. Hydrogels and tissue within the implants contain water that diffuses rapidly, however less than in the ventricles. Scale bar: 5 mm.

The edges of the lesion show glial scarring in some areas as evidenced by a GFAP staining labelling astrocytes (Fig. 7). The control lesioned rat has a dense glial scar on the right lesion edge, a palissadic astrocytic architecture at the bottom and no clear glial scar on the left lesion edge. This variability in scar presence and density would suggest another role for scars, which is to hold tissue together when necessary. Palissadic pattern has been shown to be permissive and guiding for cells that can then more easily reach the lesion [39]. The glial scar also contains dense and anisotropic collagen fibers leading to hyperintense areas on fractional anisotropy (FA) diffusion images (Fig. 7. C2–C4, D1, D4). The colors on FA images indicate the main direction of water diffusion. The scarring process can therefore be followed non-invasively by diffusion imaging. The PEGDA rats present a dense glial scar with a hyperintense rim all around the lesion (Fig. 7. A1, D1, and Supp Fig. 4. D1, D4), with a strong dorso-ventral orientation in some places in green (Fig. 7. C1-4 and Supp Fig. 4. D3). The scaring is absent or thinner for the PEGDA-GelMA and PCL rats and palissadic astrocytes associated with restorative processes were observed (Fig. 7. A2, A3, green arrows). No anisotropic diffusion areas were seen around these implants suggesting few fibrosis (Fig. 7. C2-3, D2-3). A glial pathway can be seen through the corpus callosum towards the lesion edge (Fig. 7, A2, B2, yellow arrows). However, no glial scar formed at the end of this pathway in contact with the implant. Within the implant, few GFAP staining was visible suggesting few colonization by astrocytes. Finally, mean water diffusivity images allowed to distinguish areas of low diffusivity like the abscess (Supp Fig. 4. D5) from areas with high water diffusivity (Fig. 7. E2, E3) like the implant areas with tissue reconstruction.

### 3.5 Tissue regeneration

The role of the scaffold is to provide a niche conducive to cell survival and tissue reconstruction. These biodegradable/bioresorbable scaffolds must shelter the fragile neoformed tissue during its reconstruction while slowly degrading until it disappears completely, to leave space for the consolidated neo-tissue. To that end, the implants are porous to allow cell migration. The PEGDA-GelMA implant was colonized by host cells that have adhered to the patterns and organized in oriented bundles (Fig. 6F).

The cells were counted on 14 lacunae (for the PEGDA-GelMA implant) or wire edges (for the PCL implant). On average, there were 136 ± 33 cells per lacuna and 285 cells per wire turn. This average can be normalized by the porosity of PEGDA-GelMA (27 %) and PCL (91 %) to obtain a **cell invasion factor**: 503 ± 124 for PEGDA-GelMA and 313 for PCL. Thus, PEGDA-GelMA and PCL implants can be characterized as biocompatible for the brain and allow good cell migration within them.

**Fig. 8:**
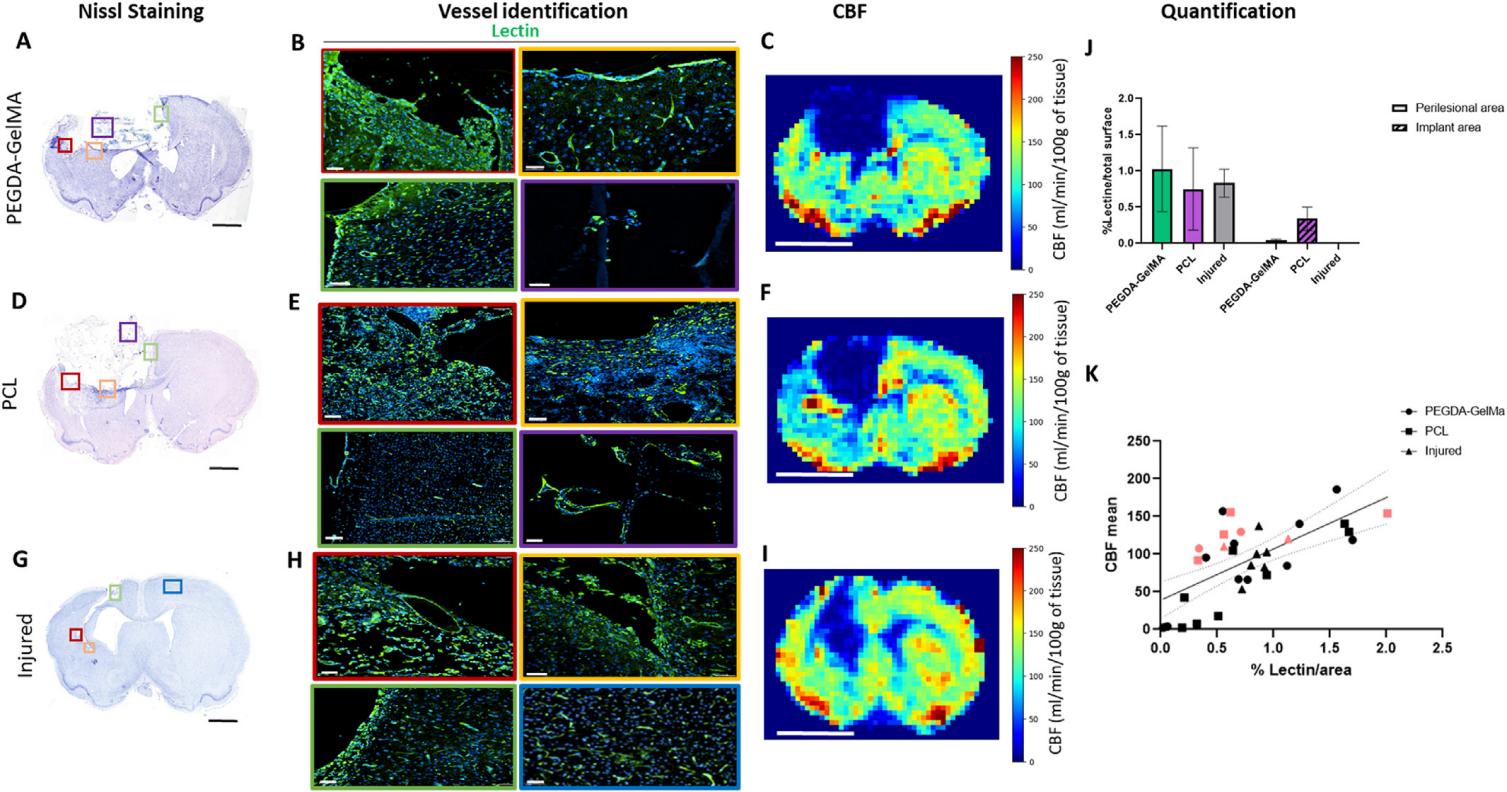
**Histology of brain sections and Cerebral Blood Flow using MRI: characterization of the vasculature**. **(A, D, G)**: Nissl staining to identity lesion border and implantation area. The colored squares indicate different areas with vascularization, in the reconstructed tissue *(red)*, at the edge of the lesion in the striatum *(orange)*, at the edge of the lesion in the cingulate cortex *(green)*, in sensorimotor cortex *(blue)* and in implant area *(purple)*. **(C, F, I)**: Cerebral perfusion (cerebral blood flow) quantitatively assessed with MRI shows the hypoperfused lesion and implantation areas. Scale bar: 5 mm. **(J)**: Perilesional and implant area vessel quantification. The entire lesion area was quantified: lectin-positive area normalized by surface area. For the implant zone, 3 fields (20x) were selected and averaged. There was no statistical difference in lectin percentage between the PEGDA-GelMA, PCL and the control injured rat. **(K)**: Correlation between CBF mean and lectin percentage per area. Points colored in pink correspond to images with large vessels. Pearson correlation gives 95 % confidence interval 0.44 to 0.83 with r = 0.68; r squared = 0.44 p∗∗∗∗<0.0001.

Vascularization was characterized with lectin staining of endothelial cells (Fig. 8B, E, H). The lesion area showed lectin-positive staining when a biomaterial was implanted in the lesion. Lectin-positive area normalized to the total surface was quantified over the entire lesion area. The normalized area of the vessels at the edge of the lesion in the rats implanted with PEGDA-GelMA and PCL was comparable to that of the injured rat (Fig. 8J). Cerebral Blood Flow was measured quantitatively with ASL (arterial spin labelling) MR imaging (Fig. 8C, F, I). The lesion and implanted areas were hypoperfused. When multiple areas were selected from different brain regions including the implant area, a positive correlation was found between lectin staining and CBF measurements (Fig. 8K). Some spots of hyperperfusion were observed around the lesion, as in the control lesioned rat, and were also observed in rats implanted with PEGDA-GelMA and PCL (Fig. 8C, F, I). Vascularization within the implants was observed by histology thanks to lectin staining; however, it was too low to appear on CBF MRI images. These biomaterials promoted endogenous neo-angiogenesis.

**Fig. 9:**
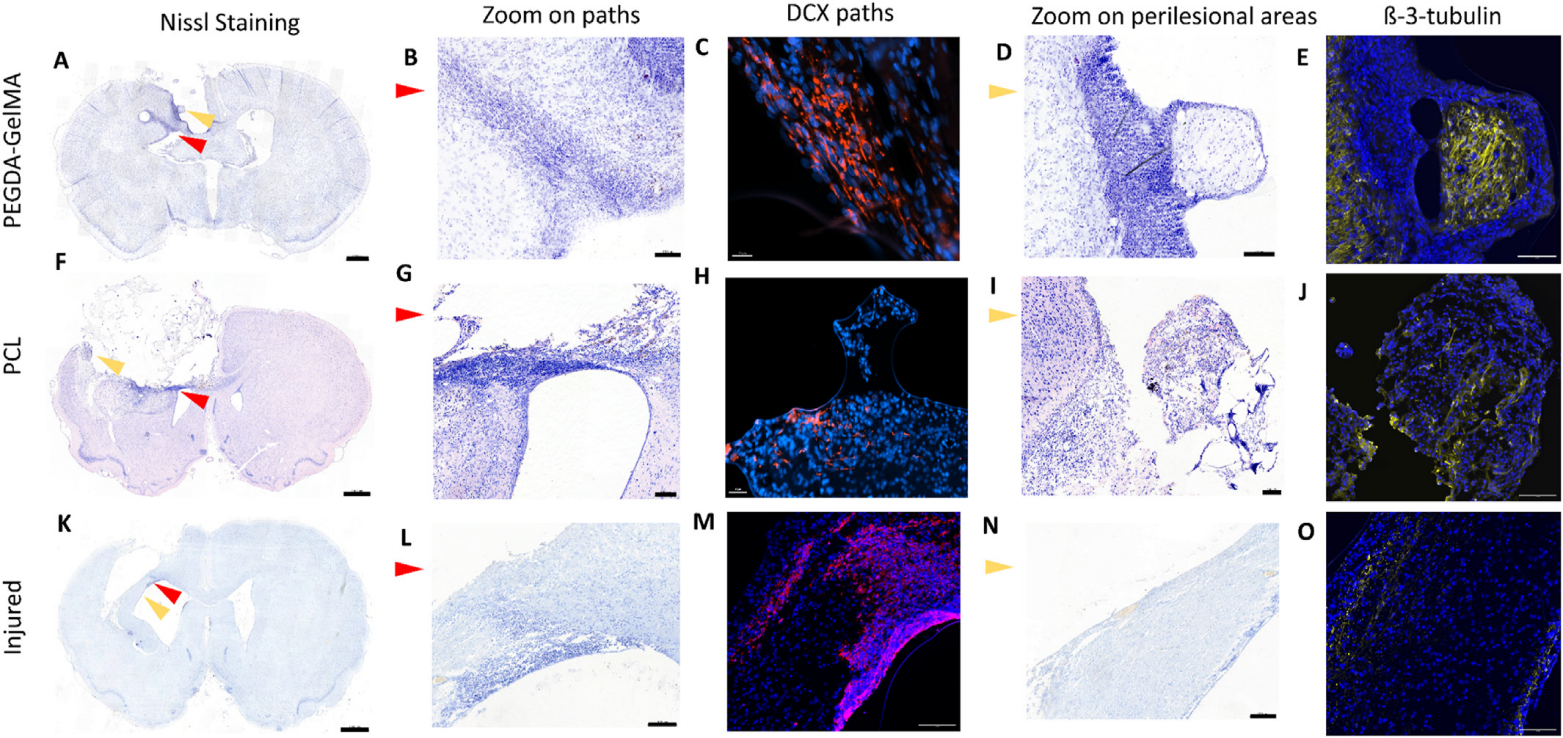
**Histology of brain sections and characterization of migration pathway**. **(A, F, K)**: Brain section at 1 month stained with Nissl Staining. **(B, G, L)**: Zoom on the paths indicated by the red arrows in A, F and K. **(C, H, M):** Doublecortin (DCX) immunofluorescent labelling of immature neuron pathways shown in B, G, and L. **(D, I, N)**: Zoom on the areas located with the yellow arrows in A, F and K. **(E, J, O)**: B-3-tubulin immunofluorescent labelling of neuronal progenitors in perilesional areas shown in D, I, and N. Scale bars: 1000  μm (histology left); 100  μm (for histology zoom middle and right).

Finally, histochemistry with cresyl-violet revealed ventriculo-lesional neural progenitor pathways in both implanted and non-implanted rats one month post-lesion (Fig. 9B–G, L). These pathways appeared to be composed mostly of neural progenitors (DCX + cells) (Fig. 9C–H, M). Implanted rats displayed a perilesional cellular environment favorable for regeneration of lost tissue. When compared to the control lesioned rat, the implantation of biomaterials in the core of the lesion provided an additional attraction for neural cells. Compared to the non-implanted rat, PEGDA-GelMA and PCL rats exhibited a higher concentration of β-3-tubulin, a marker of immature neurons, in localized areas at the lesion edge (Fig. 9E–J, O). The PEGDA-GelMA rat, in particular, had β-3-tubulin positive nerve tissue patterns embedded within the implant (Fig. 9 E).

## 4 Discussion

In this study, we investigated the regenerative potential of different bioresorbable biomaterials after a brain lesion. Out of the three 3D-printable materials tested, namely PEGDA-GelMA, PEGDA and PCL, we found that two showed promising signs of biocompatibility: PEGDA-GelMA and PCL. While PCL and PEGDA-GELMA induced a low inflammatory reaction in the brain, allowed neo-angiogenesis, and attracted new neurons, the PEGDA-GelMA implant was the one with the lowest inflammatory reaction. On the other hand, PEGDA (PEGDA-200 and PEGDA-500) induced a strong foreign body reaction in the brain.

Other studies have investigated the use of scaffolds with macroporous structures, 3D orthogonal cylindrical pores or 3D orthogonal beams, to be implanted in the brain, spinal cord or bone [14,[40], [41], [42], [43], [44]]. Yet, a complex structure with such a high spatial resolution as the one we designed had never been implanted in vivo (Fig. 2A–C). We have selected PEGDA as a base material that could be a promising candidate for brain implants since it showed various desirable properties: it is stiff enough to be manipulated, it can be easily printed on Cellink devices, with high spatial resolution and thin features, and offers adequate degradation kinetics, extending over the course of several months. Our scaffolds had a porosity close to 50 %, beams with a diameter about 150 μm, guidance in three directions, and a structure with various patterns and features, such as layers and pillars, which are essential for a tissue as complex as the cerebral cortex. The structure was designed to meet functional needs, i.e., to enable 3D tissue reconstruction in the image of the multilayered architecture of the cerebral cortex. The purpose of this conformation is to support the regeneration of organized and functional tissue after degradation of the biomaterial. It should be noted that the implants were 5 mm large. While it is possible to print more porous and thinner structures, with two-photon lithography and direct laser writing techniques for instance, these are generally limited in size (only up to 300 μm large) [45]. Due to time constraints, the implants made with PEGDA-GelMA had a simpler architecture than those with PEGDA alone, thus providing less guidance for the cells. The much more random structure of the PCL implant was not designed for straight axonal guidance but maximized porosity.

Behavior analysis was used to monitor clinical signs in implanted animals. These tests were validated by the team's previous experiments and assessed the recovery of the rats according to the treatments [27,28]. There was no worsening of the deficit associated with implants and no clinical side effects related to the biomaterials were observed, suggesting that the foreign body reaction subsequent to degradable biomaterials was well circumscribed in the brain. In the case of one animal (PEGDA-200 rat) probably infected during the surgery, the infection was concentrated in the implanted area.

Implanting a foreign body inside an organ such as the brain may constitute an aggression, which naturally triggers healing mechanisms. During this reparative process, two distinct phenomena occur: fibrosis and regeneration of the tissue. An inflammatory response is to be expected, no matter the material introduced. However, even implants that degrade very little, or very slowly, will also gradually induce an inflammatory reaction characterized by the prevalence of macrophagic cells, known as granulomatous-type inflammation [46]. Rather than finding a material that does not cause inflammation, the main aim of this study was to develop an implant with an optimal shape, composition, and size, to minimize the fibrotic response, generate a thin, permissive glial ‘scar’ and provide the best possible cellular support for the regeneration phase.

To our knowledge, few studies if any explored the brain implantation of structures made of PEGDA alone. Some studies testing PEGMA (PEG with methacrylate end groups) of high molecular weight (4600 Da) implanted in rat brains showed good acceptance [18] and others testing copolymeres of PEGMA and PLA in rat and non-human primate brains revealed slight inflammatory reactions [47,48]. Previous experiments of sub-cutaneous, intraocular, and pancreatic implantations nonetheless suggested strong biocompatibility [[49], [50], [51], [52], [53], [54]]. However, our in vivo experiments revealed signs of poor compatibility and rejection. In addition, even prior coating did not prevent the foreign body reaction, reinforcing the idea that these CellInk PEGDA-200 and PEGDA-500 are poorly suited for the brain at all. It should be noted that the coating used for PEGDA implants is known to be safe and biocompatible [27]. The brain tissue bordering these implants was mainly filled with phagocytosing macrophages and multinucleated, horseshoe-shaped giant cells characteristic of a foreign body reaction. This result is reminiscent of a strong PEGDA-induced response observed in adipose tissue, skin or liver and of a microglial reaction to PEGDA-PLGA (polylactic acid) microparticles in the brain [48,[54], [55], [56]]. Some authors reported that inflammatory reaction can increase with the degradation speed of the material, its rigidity, protein coating, or the size of its micropores, stressing that these parameters must be controlled [[56], [57], [58]]. We tested two different rigidities for PEDGA structures, yet both showed poor biocompatibility. Histological analyses and MRI imaging further confirmed that PEGDA-200 and -500 are not the best biomaterials to consider for future brain biomaterials, since they caused major inflammatory reactions and biomaterial encapsulation even with coating.

Remarkably, when PEGDA was mixed with GelMA, namely PEGDA-GelMA, the implants showed more promising results, with incorporation of the biomaterial into the surrounding tissues, cell migration within 3D structures and impressive tissue regeneration, as visible on histology. Similar responses were found with PCL implants. Importantly, however, the PCL implant showed slight tissue fibrosis around its threads. Previous experiments described an inflammatory reaction with PCL implants [14,[59], [60], [61]]. It is also important to note that the PEGDA-GelMA and PCL implants, unlike the PEGDA implants (200 and 500), were not coated with proteins. The motivation behind this choice was to see how brain tissue responded to raw materials. Mixing GelMA to PEGDA adds proteins and may render the material less stiff and lessen the host reaction [58]. In addition, we noted during brain extraction that PCL adherence to the bone flap was stronger than to the brain tissue. The biocompatibility of PCL appears to be better with bone tissue. This observation is consistent with studies of bone regeneration using PCL electrospun fibers or scaffolds [44,62,63]. On the contrary, the PEGDA-GelMA implant showed no attachment to the bone flap and remained well anchored within the lesion. It appears that this biomaterial allowed development of strong connections with the surrounding tissues. This analysis confirms the hypothesis that gelatin, added to PEGDA, could maintain viability and promote cell adhesion [8,12,13,64]. Our results are corroborated by a study testing a similar 3D printed biomaterial, PEGDA-GelMA, that served as a basis for regeneration. The biomaterial, loaded with neural progenitor cells, was implanted in the spinal cord and promoted neuronal regeneration to a greater extent than hyaluronic acid and agarose [13]. Although not in rats but in mice and not in a brain lesion as large as in the present study, another group tested PEGDA as a basis biomaterial that was mixed with hyaluronic acid. They demonstrated neuronal colonization of the cortical lesion [65]. Besides, since the regenerated tissue in the core of the implant contained few astrocytes, PEGDA-GelMA may be used in cases where astrocyte load needs to be limited.

Interestingly, PEGDA, PEGDA-GelMA and PCL structures could be identified easily on MRI high resolution T2 images. Since PEGDA and PEGDA-GelMA are hydrogels, they thus appear hyperintense on T2 imaging. As the free-water content of these hydrogels was higher than that of the edematous neural tissue at the lesion site [66], the pores were darker than the hydrogel, allowing structures to be visible on T2 images. To our knowledge, this is the first time that thin, complex structures have been so clearly observed with MRI. It should be emphasized that standard T2 MRI was used, without contrast agents or sophisticated MRI techniques [67]. Diffusion imaging also showed greater water diffusion in the pores than in the hydrogel scaffold. PCL did not contain water and was therefore dark, allowing MRI to evidence its fingerprint. Considering these findings, we recommend the use of conventional high-resolution MRI as a non-invasive method for monitoring the evolution of these biomaterials.

One strength of our study was the high correlation that was found between lectin staining and MRI perfusion assessment. This original result shows that ASL MRI imaging is therefore impressive in quantifying and predicting cerebral blood flow and perfusion, especially as it does not require the injection of a contrast agent. This is a promising sign of its undeniable usefulness for non-invasive monitoring of the progressive revascularization of regenerative implants.

One limit of the study is that a single rat was implanted with PCL because of a printer failure but we propose to report the results of this pilot experiment for comparison with other biomaterials.

In conclusion, complex structures supporting cerebral regeneration can be successfully printed from biomaterials. Biodegradable 3D implants offer a new perspective in the field of medical bioengineering. Our study showed that the choice of a suitable material, depending on the recipient organ, remains very challenging. Nevertheless, 3D printing is a very promising solution. However, the range of materials lags behind the availability and accessibility of printers: few biomaterials are commercially available, and among them, few have been tested in the brain. Although they are expected to offer the same regenerative and guiding performance as non-degradable brain implants despite their degradable nature, their role is to compete favorably by making this therapeutic strategy as minimally inflammatory as possible, with the ultimate goal of reconstructing tissue entirely of biological origin [27]. Fortunately, MRI has proved ideal for non-invasively monitoring their degradation in vivo, particularly for hydrogels such as PEGDA and GelMA. Moreover, perfusion MRI seems promising for following vascularization in chronic studies.

## Author contributions

JC, NC and MC: experimental work, analysis and interpretation of the data, data curation, and draft and editing of the manuscript. WL: experimental work, analysis and interpretation of MRI data. LR: experimental work. AB: Analysis and interpretation of MRI data. CC: funding acquisition and editing of the manuscript. FD: MRI expert, experimental work, and analysis and interpretation of the data. IRL: histopathologist expert, analysis and interpretation of the data, draft and editing of the manuscript. IL: study conception and design, analysis and interpretation of the data, draft and editing of the manuscript, funding acquisition, and project administration. All authors contributed to the article and approved the submitted version.

## Funding information

This work was supported by Foundation “Gueules Cassées” [grant numbers 70-2019, 23-2019, 24-2020, 24-2021, 55-2021, and 22-2022], Agence Innovation Défense [JC], and National Research Agency (ANR) [Grant number ANR-19-ASTR-0027]. This work, bearing the reference EUR CARe N°ANR-18-EURE-0003, has benefited from State aid managed by the Agence Nationale de la Recherche under the Programme Investissements d'Avenir.

## Declaration of competing interest

We have no conflict of interest to disclose.
