# Supplementary material for: In vivo biocompatibility assessment of 3D printed bioresorbable polymers for brain tissue regeneration. A feasibility study
Source: Regen Ther. 2024 Oct 23;26:941–55. doi: 10.1016/j.reth.2024.10.004 (PMC11541680; doi:10.1016/j.reth.2024.10.004)
Supplement: Multimedia component. 1 [file mmc1.docx]

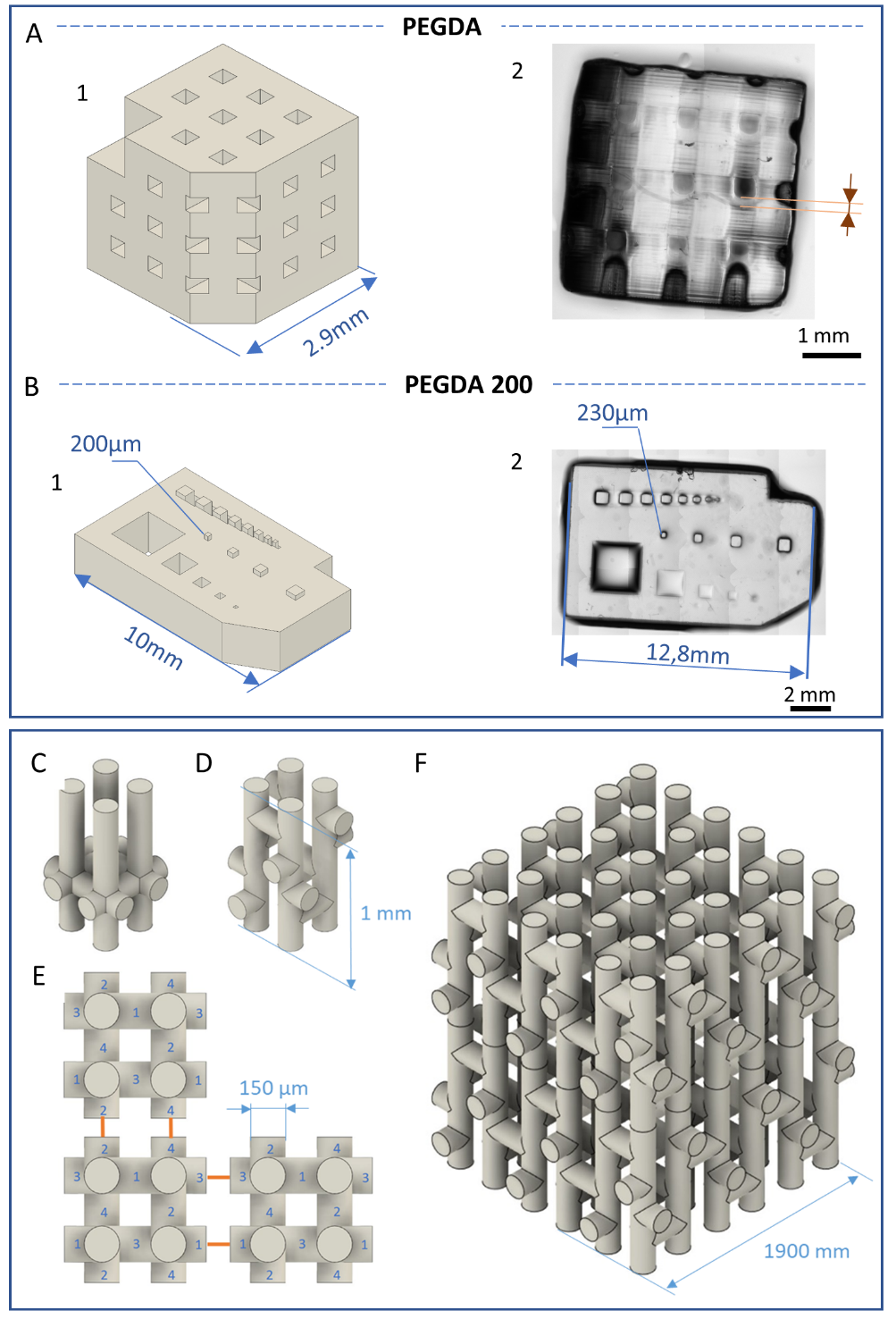


Supplementary Figure 1: **Pilot CAD and 3D printed architectures.** (A) 1: This CAD (Computer Aided Design) allows us to measure the depth of photo-crosslinking and ensure the structure has open holes. 2: Microscopic view of the printed structure (brightfield). The structure is roughly reproduced. The pores are visible, and the lines indicate the horizontal print layers. The printer will print a new layer every 50 µm. However, this does not mean that the curing depth is also 50 µm. In the case where there is a void behind the currently printed layer, the light does not stop at 50 µm and the layer will be thicker than 50 µm. We can see an example of that between the two-orange lines. This is a single layer, and yet it is 100 µm thick instead of 50 µm. If the curing depth is too great, the channels can be completely clogged. (B) 1: CAD design used to measure the difference between the feature size from the model and the one from the structure printed. 2: Microscopic view. When using PEGDA-200, the 200 µm pillar was actually printed at 230 µm. By measuring this structure, we can determine the minimal printable size for the features and adjust the CAD design dimensions to match the desired outcome. (C-E) 3D pattern to reduce buckling. (C) Classic pattern not chosen (D) Modified pattern with the same number of horizontal beams but an increased resistance to buckling. To improve rigidity, we eliminated the planes of symmetry and minimized beams length. (E) To adjust the implant size to the lesion size, we repeated the original pattern in all three dimensions. The beams are distributed over four levels (indicated in blue) so that the pattern is compatible with itself. (F) Resulting structure with a 3x3x2 matrix of the pattern B.


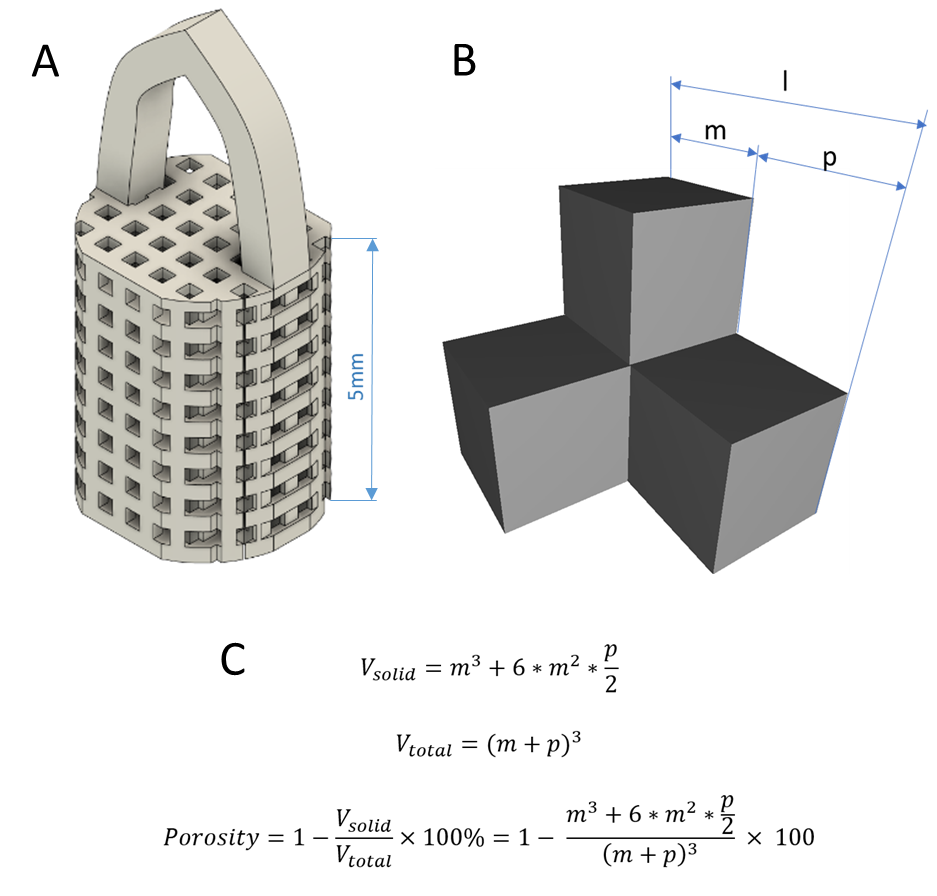


Supplementary figure 2: **Porosity calculation for PEGDA-GelMA structure.** (A) 3D geometry of the implant. The handle is removed during surgery. (B) Elementary pattern repeated in all three dimensions to produce the 3D geometry of the implant. l is the size of the cubic pattern, m is the width of the pillars, and p is the width of the channels (C) Equations used to calculate porosity.


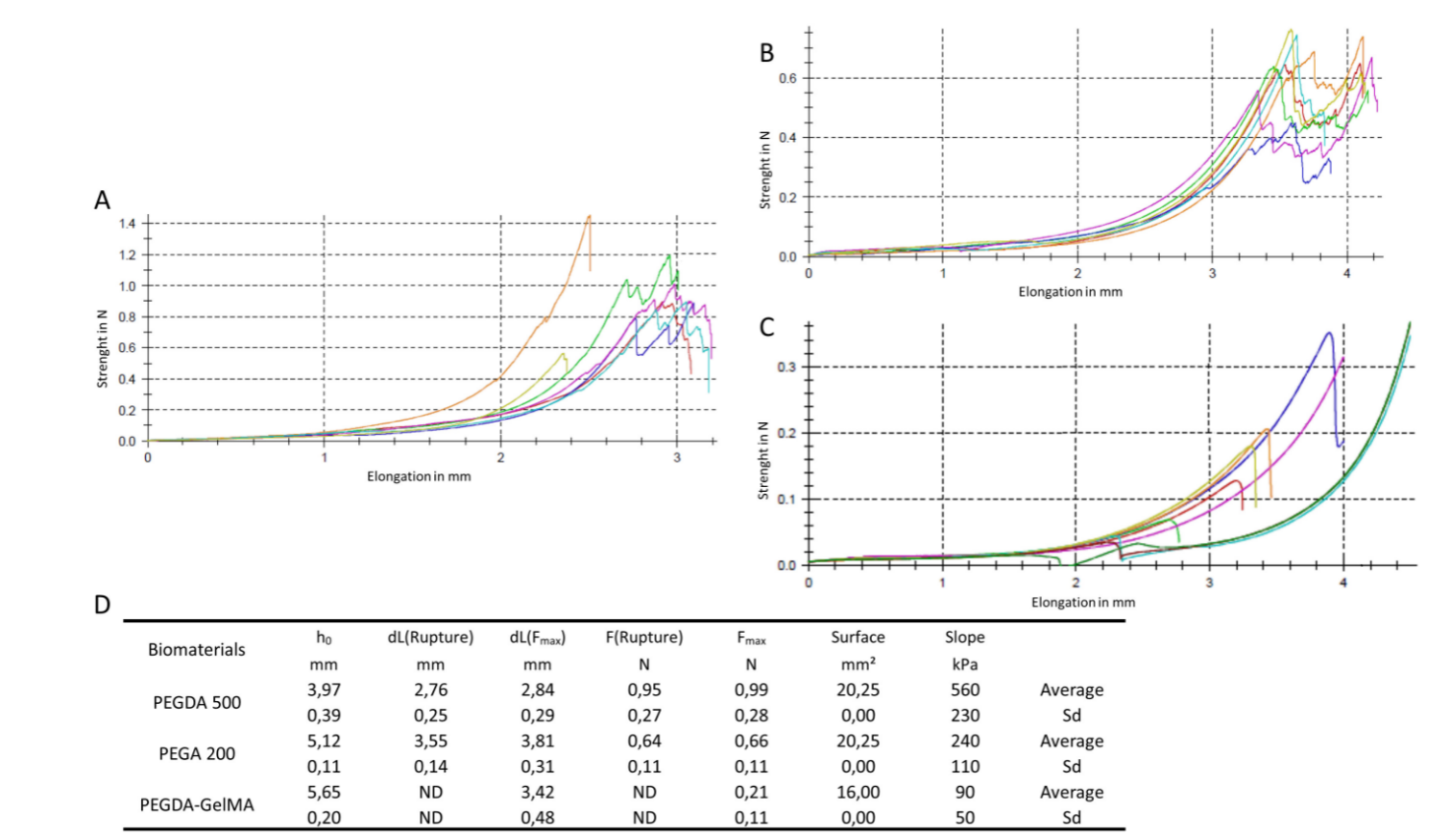


Supplementary Figure 3: **Mechanical characterization of the structures**: unconfined compression on vertical samples until failure. A/B/C: Stress-strain curves obtained for respectively PEGDA 500 on 8 samples; PEGDA 200 on 7 samples; PEGDA-GelMA on 6 samples (The curves on the right represent the stress-strain curves obtained on 3 samples that tipped over in a horizontal position. They were not included in subsequent calculations). It should be noted that the bio-implants, not having a perfect geometric shape, sometimes showed a slippage during measurements. D: Table showing characteristic values for the three biomaterials PEGDA 500, PEGDA 200 AND PEGDA-GelMA.

**
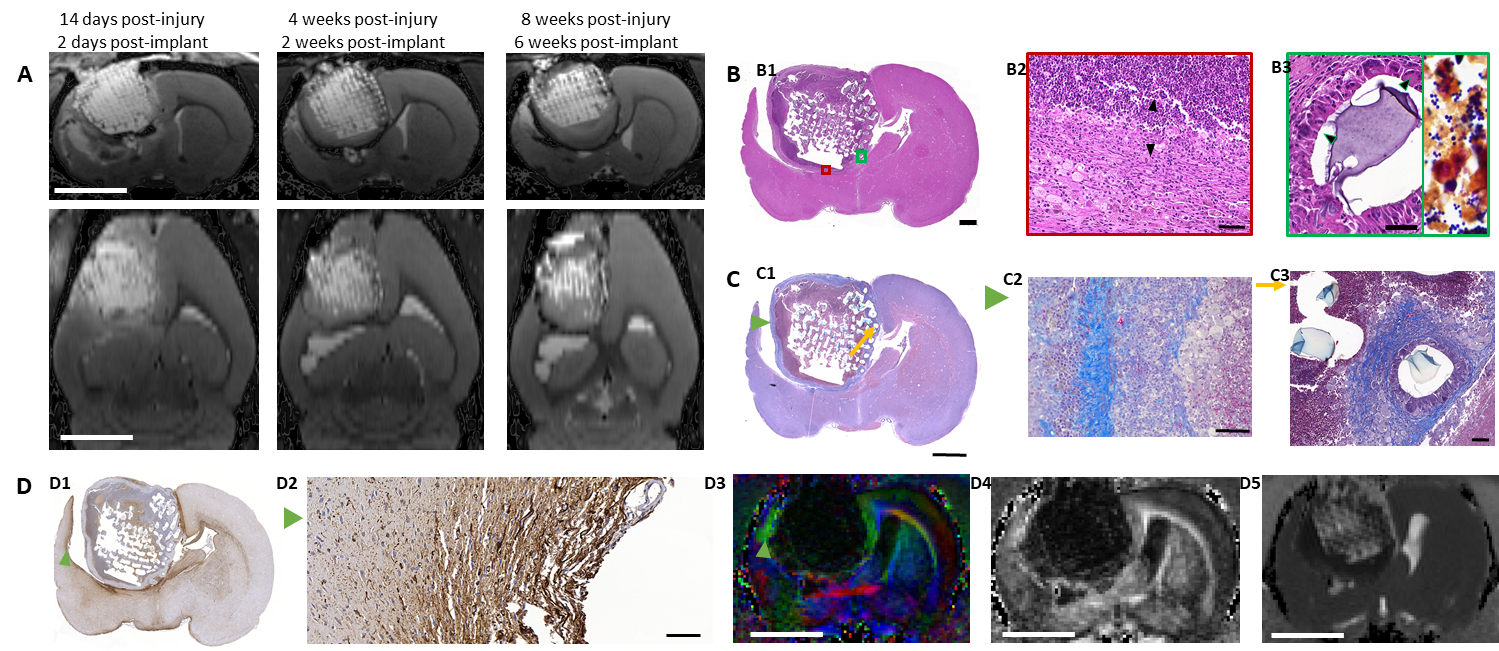
**

Supplementary figure 4: **MRI and histology of the infected PEGDA 200 rat.**

A : Longitudinal MRI follow-up before and after implantation of a scaffold in the brain lesion. Coronal and axial T2 MR images of the lesion and the implants. PEGDA-200 was encapsulated in a hypointense fibrosis scar in a rat infected by a staphylococcus. Scale bar: 5 mm.

B : Histological sections of PEGDA 200 rat brain. (B1) Brain section at 2 months stained with HE, bregma -0.60mm. Implant encapsulated and well delimitated from the rest of the brain in a large infectious abscess. (B2) Tissue dissociation between an internal basophilic suppurative area (degenerated and pyknotic neutrophils) (arrow up) and a more eosinophilic, less dense area of residual brain tissue containing large foamy phagocytic macrophages, hemosiderin and fibroblasts (arrow down). (B3) Typical foreign body reaction around PEGDA-200 residual piece encapsulated by palisading macrophages and multi-nucleated giant cells not contaminated by suppuration. Insert: Gram positive coccobacilli in the suppurative collection. *Scale bar: 1000µm (left histology); 50µm (for histology zoom middle and right) HE: hematoxylin-eosin*.

C : Histology of trichrome stained brain section and characterization of perilesional and implanted areas. (C1) Topography of lesions at low magnification on coronal sections stained with Masson’s trichrome (left). The collagen deposit is identified by the blue coloration surrounding the implants. Higher magnification shows perilesional brain tissue (green arrowhead). (C2) Collagen fibers accumulate in a thick layer around the periphery of the abscess. (C3) A fibrosing reaction is seen around PEGDA-200 with a thick central ring of macrophages surrounded on the outside by abundant collagenous fibrosis associated with other macrophages and separated from the suppuration.

*Scale bar: 1000 µm (left); 100 µm (middle); 50 µm (right).*

D: Histology of brain sections and Colored-FA, FA, MD by MRI: characterization of glial scarring. (D1) : Glial fibrillary acidic protein (GFAP) immunostaining was used to identify glial scar formation after injury, implantation and infection. The green arrow highlights (D2 zoom) the perilesional area showing glial scar formation. (D2) Astrocytes are thin and aligned perpendicular to the lesion with a palisading effect. (D3) MRI water diffusion images of coronal sections of rat brain. Colored-FA (fractional anisotropy) images show main directions of water diffusion (green: vertical; blue: antero-posterior; red: left-right). This dense scar is visible on MRI. (D4) FA images show anisotropic water diffusion regardless of direction. (D5) MD (mean diffusivity) quantifies water diffusion speed in mm²/sec. Hydrogels and tissue within the implants contain water that diffuses rapidly, however less than in the ventricles. Scale bar: 5 mm.

To summarize, the implant was fully encapsulated in a hypercellularized spherical structure on histological HE sections. Macroscopic analysis showed this inflammatory mass was completely dissociated from the rest of the brain. Once removed with surgical forceps, the surrounding tissues appeared whitish, with a fibrous appearance. The implant has been rejected by the immune system and isolated from the brain tissue. Masson's trichrome staining showed the implant surrounded by blue collagen fibers. This foamy abscess was positive to GRAM staining. There was a suspicion of a staphylococcus aureus infection reported as a contaminant present in the zootechnics housing at the same time. The bacterial load was high, with gram-positive coccobacilli in the extracellular space and some in the macrophages. The infection appears to be concentrated around the PEGDA-200 implant. This immune response can also be observed with GFAP labelling: astrocytes appeared hypertrophic and formed a glial scar (brown all around the abscess, Supp Fig. 4D1-2). This protection is probably the reason why the rat’s health did not clinically deteriorate.
